# Utilizing Constrained Bicyclic Peptides for *In Vitro* Diagnostics

**DOI:** 10.1021/acsnano.5c19041

**Published:** 2026-02-13

**Authors:** André Shamsabadi, Adam Creamer, Christy J. Sadler, Aida Abdelwahed, Katherine U. Gaynor, Yuliya Demydchuk, Gabriela Ivanova-Berndt, Katerine Van Rietschoten, Paul Beswick, Liuhong Chen, Gustavo Arruda Bezerra, Aleksei Lulla, Paul Brear, Marko Hyvönen, Michael J. Skynner, Molly M. Stevens

**Affiliations:** † Department of Materials, Department of Bioengineering and Institute of Biomedical Engineering, 4615 Imperial College London, London SW7 2AZ, U.K.; ‡ Bicycle Therapeutics, Portway Building, Granta Park, Cambridge CB21 6GS, U.K.; § Department of Biochemistry, University of Cambridge, Cambridge CB2 1GA, U.K.

**Keywords:** bicyclic peptides, antibody mimic, ELISA, LFIA, diagnostics

## Abstract

Constrained bicyclic peptides (*Bicycle molecules*) with high affinity for biological targets have emerged as potentially powerful therapeutic agents, particularly for the *in vivo* targeting of cancer receptors. However, their antibody-mimetic properties have yet to be explored for use in diagnostic immunoassays. These synthetically derived compounds serve as biorecognition scaffolds that allow for facile site-selective modification and large-scale production. A phage display screen against various constructs of the SARS-CoV-2 nucleocapsid (N) protein identified several *Bicycle* molecules with binding affinities ranging from the micromolar to the low nanomolar range. These *Bicycle* molecules were validated in the development of enzyme- and nanozyme-linked immunosorbent assays, as well as enzymatic and colorimetric nanoparticle-based lateral flow immunoassays (LFIA) for the detection of ultralow concentrations of the SARS-CoV-2 N protein. We envision that these moieties enable robust, cost-effective, and large-scale development of ultrasensitive biosensors for a diverse range of biomarkers by leveraging their high binding affinity, minimalistic scaffold, and synthetic accessibility.

## Graphical abstract

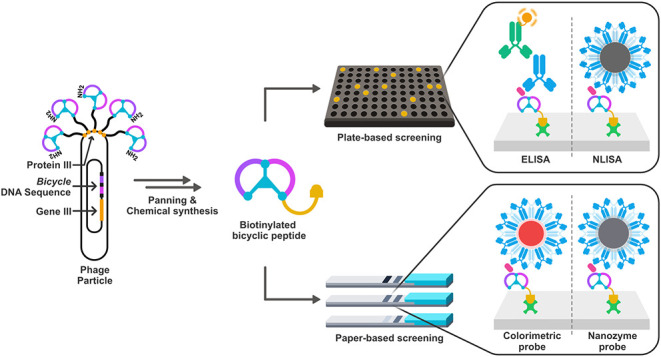

Biorecognition elements that specifically and effectively associate with disease-indicating targets are central to the function of biosensors and diagnostic technologies. While the underlying modular platforms of biosensor assays are well established, their adaptation for the detection of specific biomarkers is contingent on the target-specific nature of the binding agents used. Thus, in the development of high-performing diagnostic platforms, generating binding constructs that exhibit high affinity and highly specific targeting of relevant biomarker epitopes is of great importance.

The most common method for recognizing a biomarker target involves a pair of biorecognition elements, where the formation of a “sandwich complex” immobilizes a probe, positively indicating the presence (or, in some instances, absence) of the biomarker. The biorecognition element pair has distinct roles in the formation of these complexes: the “capture” element affixes to a surface and immobilizes the target antigen, while the “detection” element is tethered to an indicator probe. To maximize the sensitivity, both recognition elements typically do not compete for the same epitope. The premier methods of antigen detection in a laboratory setting (enzyme-linked immunosorbent assay (ELISA)) and at the point-of-care (lateral flow immunoassay (LFIA)) often rely on sandwich complex formation to achieve quantitative, semiquantitative, or qualitative detection of biomarkers. In most cases, antibodies are employed due to their adequate sensitivity (inherent binding affinity between the target antigen and antibody), general specificity (acceptable nonspecific interactions or off-target binding when utilizing monoclonal species), and established manufacturing processes. Currently, the immunoglobulin G (IgG) class of antibodies are the predominant biorecognition elements used in biosensors.^1^ However, alternative antibody mimics such as fragment antigen-binding (Fab) antibodies,^2,3^ single-chain variable fragments (scFv),^4,5^ nanobodies (Nbs),^6,7^ and aptamers^8,9^ have also been employed.

Constrained bicyclic peptides (*Bicycle molecules*) are a novel class of affinity agents that have emerged as targeted cancer therapeutics due to their high affinity (nanomolar to picomolar),^10^ absolute selectivity for targets of interest, and low molecular weight, which enables effective tissue penetration.^11^ *Bicycle molecules* are chemically constrained bicyclic peptides, typically consisting of 10–20 amino acids and have been found to exhibit binding affinities directly comparable to those of antibodies.^12^ Owing to their minimalistic architecture and lack of high-order structure, *Bicycle* molecules offer the potential for expected superior stability and significantly improved specificity (through reducing nonspecific binding interactions) when compared to traditional biorecognition elements. This is achieved by omitting superfluous protein domains that are typically present in IgGs and other antibody derivatives or mimics that are unnecessary for target recognition and may promote undesired intermolecular interactions.^13^

Furthermore, their chemical synthesis allows *Bicycle molecules* to be readily and site-specifically modified at positions distant from the antigen-binding site, a feat that is notoriously difficult with antibody-based alternatives.^14^ This characteristic is particularly desirable for the effective use of binders in immunoassays, where the orientation and unimpeded presentation of binding sites are instrumental for optimal bioassay performance.^15,16^ Additionally, the chemical synthesis of *Bicycle molecules* ensures consistent batch-to-batch production at a scale, offering a significant advantage over immunoglobulin manufacturing. These properties suggest that *Bicycle molecules* may represent a highly desirable class of biorecognition elements for biosensors in *in vitro* biomarker detection.

Here, we demonstrate the use of site-specifically biotinylated *Bicycle molecules* identified against the severe acute respiratory syndrome coronavirus 2 nucleocapsid (SARS-CoV-2 N) protein, which was chosen as the target due to its high abundance in coronaviruses, making it an optimal biomarker for disease detection. These *Bicycle molecules* were evaluated as effective biorecognition elements in enzyme- and nanozyme-linked immunosorbent assays (ELISA and NLISA, respectively). This method was extended to colorimetric nanoparticle- and nanozyme-based lateral flow immunoassays (Figure 1). While gold nanoparticles (AuNPs) are established signal transducers in the field, we also utilize platinum-coated nanocatalysts (PtNCs) here to demonstrate the synergistic effect of combining orthogonal bioassay-enhancing strategies (catalytic amplification and synthetic bicyclic peptide binders) to achieve ultralow sensitivities. This showcases the compatibility of *Bicycle* molecules with various transduction methods and immunoassay platforms, ranging from standard colorimetric readout to catalytic signal amplification. Utilizing these strategies, we established an ultralow limit of detection (LoD) for the SARS-CoV-2 N protein in both plate- and paper-based biosensor formats.

**Figure 1:**
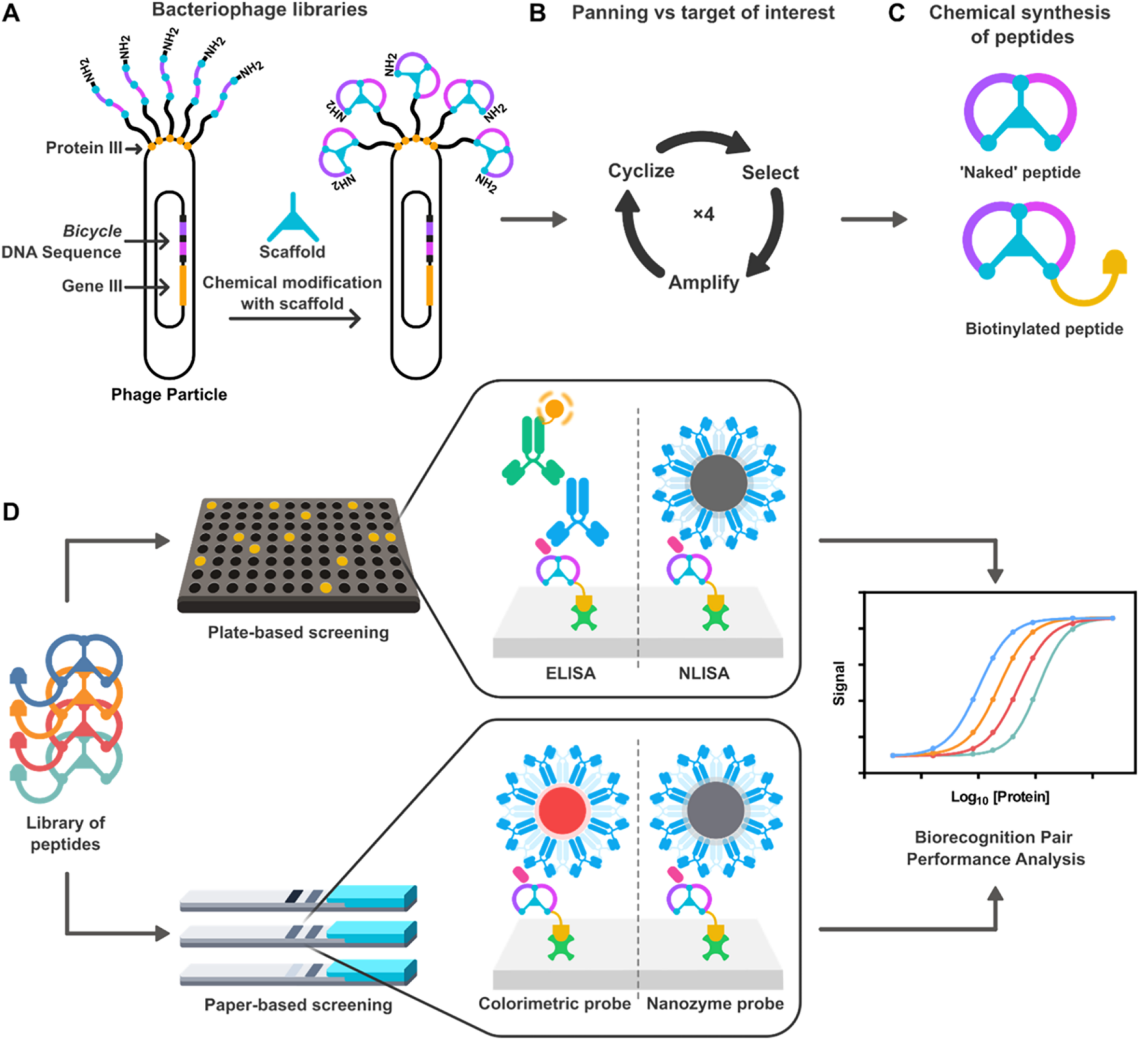
(A) Generation of *Bicycle* bacteriophage libraries: Sequences encoding *Bicycle molecules* were inserted in-frame with gene III to encode linear peptide chains as in-frame fusions with phage coat protein III expressed on the surface of the viral particle. Chemical modification with a symmetrical trivalent chemical scaffold results in covalent thioether bond-driven cyclization of the peptides to form a *Bicycle molecule*. (B) Phage selection process. *Bicycle* phage libraries were “panned” against different N protein constructs in an iterative process of selection, amplification, and further cyclization to enrich for specific protein binders. (C) Chemical synthesis of peptides: Binders identified as “hits” through phage display were chemically synthesized as peptides—in a “naked”, untagged format for initial determination of binding affinity, and in a biotinylated format to enable use in diagnostic assays. (D) Schematic showing the process for evaluating the performance of *Bicycle molecules* in plate- and paper-based immunoassays for the detection of protein targets.

## Results and Discussion

### Phage Display

To obtain novel affinity agents against the SARS-CoV-2 Nucleocapsid (N) protein, chemically biotinylated full-length, C-terminal domain (CTD), or N-terminal domain (NTD) recombinant proteins from the Wuhan-Hu-1 strain of SARS-CoV-2 (see Text S1 for full sequences) were used as target material in solution-based panning selections using *Bicycle* bacteriophage (phage) libraries. These libraries consisted of linear peptides (10–20 amino acids in length) containing three cysteines, which were cyclized *in situ* to form thioether-bonded bicyclic peptide libraries (Figure 1A).^17^ After four rounds of selection, enriched phage clones were isolated, sequenced, and screened for protein binding using an *AlphaScreen* assay. Selected affinity agents were synthesized as peptides and characterized for binding affinity determination against each protein construct using biolayer interferometry (BLI). This identified binders with micromolar to low nanomolar affinities and demonstrated that distinct *Bicycle molecules* could be readily and rapidly obtained to target different regions of the N protein (see Table S1 for *Bicycle* sequences, scaffold, and dissociation constant as determined by BLI). A range of binders were then synthesized with a biotin handle to enable exploration of optimal combinations of affinity, epitope, and sequence families in diagnostic platforms (all compounds were isolated at a >95% purity by HPLC, see Traces S1–S4).

### ELISA Screening of Sandwich Pairs for the Detection of the SARS-CoV-2 N Protein

For the sensitive and specific detection of a target antigen in ELISA, a biorecognition element pair that collegially recognizes the target antigen is required. A hybrid sandwich format (utilizing *Bicycle* capture and antibody detection probe) was selected for this proof-of-concept study. While a full *Bicycle–Bicycle* sandwich is theoretically possible, the use of biotinylated *Bicycle* molecules as a detection probe would require careful engineering to prevent cross-reactivity with the streptavidin surfaces (where a nanoparticle (poly)­streptavidin coating is employed to utilize the biotinylated *Bicycle* molecules as detection probes). To evaluate the applicability of biotinylated *Bicycle molecules* for plate-based *in vitro* protein detection, 16 biorecognition pairs obtained from the combination of four monoclonal anti-SARS-CoV-2 N protein NTD IgGs (Sino Biological, cat: **40143-MM05**, **40143-MM08**, **40588-MM123**, & **40588-MM124**) with four biotinylated *Bicycle molecules* (**B001**, **B002**, **B003** & **B004**) were trialed to identify the optimal pair for detection of recombinant SARS-CoV-2 N protein (Sino Biological, cat: 40588-V08B). The orthogonality of formed capture *Bicycle molecule*–detection IgG sandwich complexes allowed for the use of an enzyme-tagged anti-IgG secondary antibody as a universal probe in all ensuing experiments. Initially, a signal-to-noise screening experiment was carried out to observe combinations of capture *Bicycle* molecule and detection IgGs that could successfully recognize the antigen (signal) with minimal background (noise, signal produced using a blank sample) at a clinically appropriate intermediate amount of target antigen.^18^ At 50 ng·mL^–1^ of the N protein, most trialed combinations produced an observable signal above their corresponding background (analogous experiment carried out with no antigen present), albeit to varying extents (Figure 2A). See Figures S1 and S2 for the corresponding standard deviation and coefficient of variation values, respectively. Notably, *Bicycle molecules* binding to N protein CTD (**B003** and **B004**) appeared to have superior performance, providing higher S/N ratios compared to *Bicycle molecules* generated against the N protein NTD. This is perhaps to be expected, as the trialed monoclonal IgGs were all raised to target the N protein NTD; however, certain pair combinations consisting of two elements against the NTD were also found to provide significant signal above their background. We can therefore speculate that the binding epitope of *Bicycle molecules* in these pairs does not overlap with those of their corresponding IgGs, even if they both interact with the same domain. To demonstrate the binding of **B003** and **B004** to the N protein CTD, BCY00018176, a Bicycle molecule belonging to the same family, was crystallized, and its structure was solved. The N protein CTD domain is responsible for N protein dimerization. Accordingly, the verification of two molecules in the asymmetric unit corresponds to a stable dimer, as previously described.^19^ Two Bicycle molecules interact symmetrically with residues D341-N354 from one subunit and residues M322-T329 from the adjacent subunit (Figure S3). The binding location of Bicycle molecules from the **B001** and **B002** series was also confirmed by crystallizing BCY00017628 in complex with N protein NTD. The NTD region of the protein interacts with viral genomic RNA to facilitate viral transcription and assembly. Bicycle molecule interact with residues R68, D81, I84, Y123, and A134-N140 (Figure S4).

**Figure 2:**
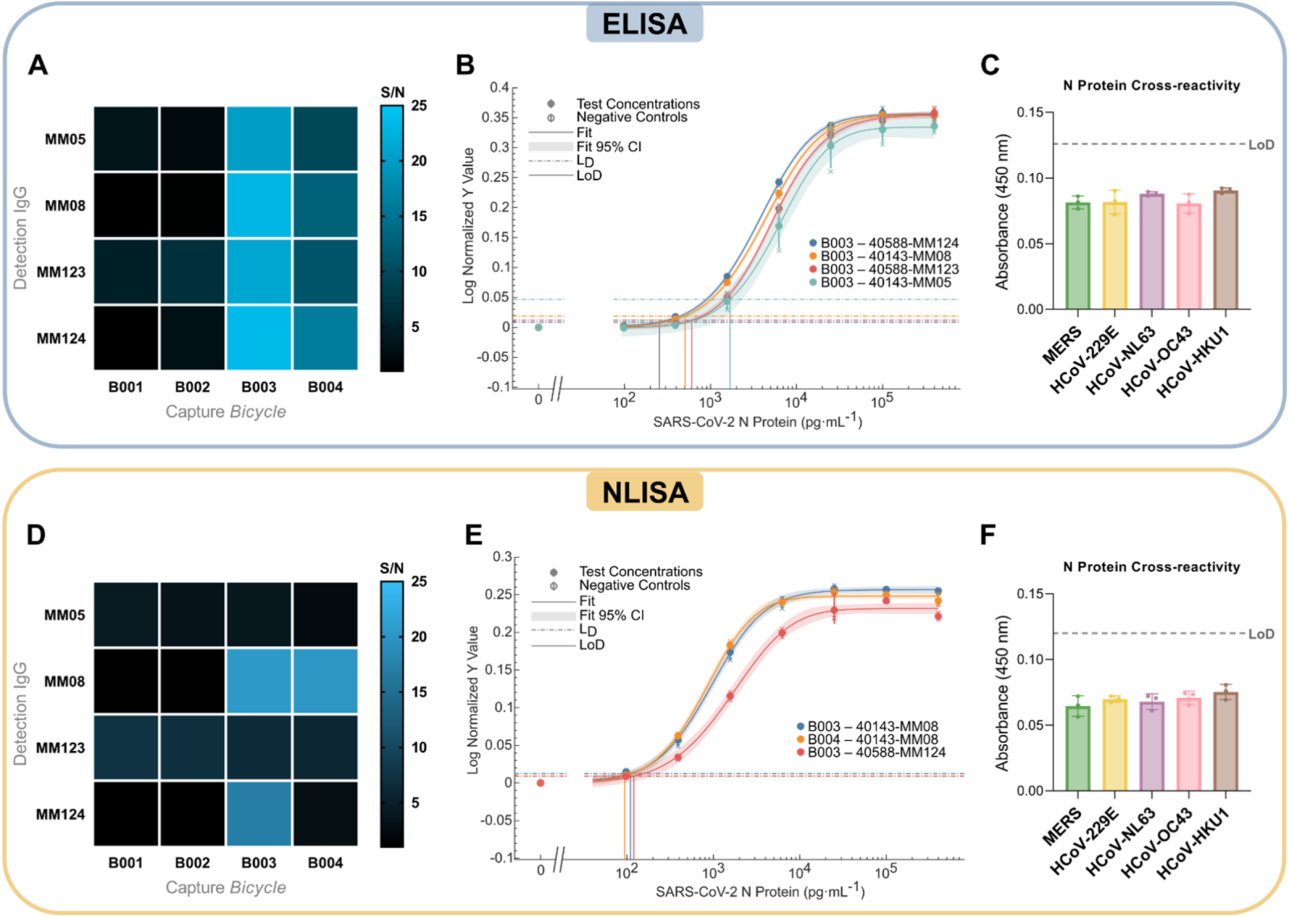
(A) Biorecognition pair ELISA signal-to-noise matrix heatmap at 50 ng·mL^–1^ SARS-CoV-2 N protein, *n* = 3. (B) ELISA calibration curves of the best performing pairs for the detection of the SARS-CoV-2 N protein, data shown as mean ± S.D, *n* = 3. (C) ELISA cross-reactivity study for 1 μg·mL^–1^ of HCoV N proteins, data shown as mean ± S.D, *n* = 3. (D) Biorecognition pair NLISA signal-to-noise matrix heatmap at 10 ng·mL^–1^ SARS-CoV-2 N protein, *n* = 3. (E) NLISA calibration curves of the best performing pairs for the detection of the SARS-CoV-2 N protein, data shown as mean ± S.D, *n* = 3. (F) NLISA cross-reactivity study for 1 μg·mL^–1^ of HCoV N proteins, data shown as mean ± S.D, *n* = 3. *L*_D_ refers to the decision limit, which is calculated as the mean of the blank plus three times the standard deviation of the blanks (*S*_0_ + 3σ_*S*0_), whilst LoD refers to the limit of detection as calculated *via* a statistical method derived from Holstein et al.,^20^ which considers the variance of the blank samples and test samples.

Nevertheless, it was observed that all four combinations utilizing **B003** as the capture biorecognition element provided the greatest S/N for the detection of 50 ng·mL^–1^ of the recombinant SARS-CoV-2 N protein. Further testing of these four pair combinations using different concentrations of N protein demonstrated typical dose–response curves (Figure 2B). Limit of detection (LoD) values are calculated utilizing a robust statistical method derived from Holstein et al.,^20,21^ which considers the variance of the blank samples and test samples and is considered a pragmatic method for the calculation of detection limits compared to other widely reported methods. Tables S2–S9 show all the detection limit fitting parameters from this method for each combination of capture and detection probes and four-parameter logistic regression best-fit values. Utilizing this, it was predicted that biorecognition pair **B003–40588-MM124** would have the greatest potential in detecting the SARS-CoV-2 N protein at lower concentrations, owing to the pair providing the lowest calculated LoD (see Table S10 for the ANOVA test between trialed pair combinations). Thus, the **B003–40588-MM124** pair was chosen as the ultimate pair to generate a comprehensive ELISA calibration curve and conduct a cross-reactivity study against the N protein of other known human coronaviruses (HCoVs). A checkerboard titration ELISA was also performed to calculate the working operational concentrations of the **B003–40588-MM124** pair (see Figure S6). With optimized conditions, a final LoD of 250 pg·mL^–1^ for the SARS-CoV-2 N protein was observed. The decision limit, calculated as the mean of the blanks plus three times the standard deviation of the blanks (*S*_0_ + 3σ_*S*0_), was calculated to be 180 pg·mL^–1^ (see Figure S5 and Table S11 for the sigmoidal regression curve and detection limit fitting parameters). No cross-reactivity was observed with respect to 1 μg·mL^–1^ of the other HCoV N proteins (Figure 2C).

### NLISA Screening of Sandwich Pairs for the Detection of the SARS-CoV-2 N Protein

NLISA is an increasingly popular technique for *in vitro* protein detection, where nanozymes emulate the role of the enzymatic probe utilized in ELISA formats.^22^ The omission of the biological enzyme probe in place of nanozymes typically allow the NLISA format to achieve superior sensitivity with respect to ELISA (owing to use of probes with higher catalytic constants (*k*_cat_)), and allows for greater biorecognition element compatibility, where covalent bioconjugation techniques to affix the enzymatic probe or the use of secondary antibodies which need to selectively recognize the detection probe can be avoided.^23^ Thus, a process similar to that of the ELISA development *vide supra* was then repeated to demonstrate the feasibility of biotinylated *Bicycle molecules* in NLISA format.

Peroxidase-mimicking platinum-based nanocatalysts (PtNCs) were utilized as detection probes in the NLISA format. The PtNCs consisted of a 15 nm gold seed with a porous platinum shell, synthesized following our previously reported procedure^24^ (see Figures S7–S10 for DLS and TEM of gold seed and PtNC). Here, the aforementioned monoclonal IgGs utilized as the detection biorecognition element in the ELISA experiment were adsorbed onto the PtNCs (see Figure S11 and Table S12 for DLS and ζ-potential of bare and IgG-coated PtNCs). Akin to the preliminary screening in the ELISA study, a signal-to-noise screening experiment was conducted to determine which combinations of the capture Bicycle molecule and detection IgG would result in the greatest signal generation with the least background. Owing to the expected increased sensitivity of the assay, the screening was therefore conducted at 10 ng·mL^–1^ of recombinant SARS-CoV-2 N protein. All pair combinations of the biorecognition elements were tested, and it was again observed that, to varying degrees, almost all pairs produced an observable signal above their corresponding background (Figure 2D). See Figures S12 and S13 for the corresponding standard deviation and coefficient of variation values, respectively. As expected, pair combinations that provided significant signal-to-noise in the ELISA screen were also consistent in producing good signal-to-noise ratios in the NLISA format, whereas pairs that did not perform well in ELISA were also found to not be suitable for the detection of the SARS-CoV-2 N protein in NLISA. However, the relative performance of these biorecognition element pairs did not seem to directly translate between the two formats, with some pairs performing significantly better in one assay over the other and *vice versa*.

Nonetheless, three superior pairs were observed, and further testing of these combinations at different concentrations of recombinant SARS-CoV-2 N protein yielded typical dose–response curves (Figure 2E). As above, Tables S13–S18 show all the detection limit fitting parameters from this method for each combination of capture and detection probes and four-parameter logistic regression best-fit values. **B004–40143-MM08** was ultimately chosen as the pair combination that would have the greatest potential to detect N protein at lower concentrations owing to the pair displaying the lowest calculated LoD, although the detection limit performance was largely indistinguishable from the other trialed pairs (see Table S19). Calculation of LoD values of the generated curves resulted in the **B004–40143-MM08** biorecognition pair being chosen as the ultimate pair to generate a comprehensive NLISA calibration curve and a cross-reactivity study against the N protein of the other known HCoVs. A checkerboard titration NLISA was also performed to calculate the working operational concentrations of the **B003–40588-MM124** pair (see Figure S15). With optimized conditions, a final LoD of 97 pg·mL^–1^ and a decision limit of 23 pg·mL^–1^ for the SARS-CoV-2 N protein was observed (see Figure S14 and Table S20 for sigmoidal regression curve and detection limit fitting parameters). No cross-reactivity with respect to 1 μg·mL^–1^ of other HCoV nucleocapsid proteins was observed (Figure 2F).

### *Bicycle Molecules* in Paper-Based Immunoassays

Paper-based diagnostics are the current flagship bioassay platform for PoC (point-of-care) detection of disease-indicative biomarkers.^25^ This was aptly demonstrated during the COVID-19 pandemic, which highlighted that in the context of infectious diseases, the rapid diagnosis and isolation of infected individuals were important methods to control virus transmission only made possible through the use of widely distributed lateral flow tests.^26^

Notwithstanding, lateral flow tests have the potential for the detection of biomarkers associated with noncommunicable diseases and markers in physiological states, which is illustrated by the widespread use of lateral flow devices in the detection of human chorionic gonadotropin for the determination of pregnancy. With the performance of *Bicycle molecules* as an effective capture biorecognition element in plate-based immunoassays in hand, our attention was turned to validating the use of biotinylated *Bicycle molecules* in paper-based diagnostic platforms. Analogous to plate-based diagnostic platforms, a biorecognition element pair capable of sensitively and selectively recognizing its appropriate target is a requisite for paper-based diagnostics.

### Nanozyme LFIA

Compared to standard colorimetric LFIA, which typically use gold nanoparticles (AuNPs) as the indicator probe, the use of peroxidase-mimicking nanozymes can achieve ultralow visible limits of detection as catalyzed oxidation of a colorimetric substrate results in a signal-amplified readout. The resulting increase in the test line intensity allows for the identification of visible test lines that would otherwise go undetected with the use of typical colorimetric nanoparticle probes.

To determine which biorecognition pairs would perform best in LFIA, we initially conducted preliminary screening involving all 16 *Bicycle*-IgG pair combinations. Although a previous biorecognition pair screen was conducted using catalytic nanozymes as the detection probe in the context of NLISA, it was thought appropriate to conduct an independent preliminary screening experiment of the biorecognition pairs for LFIA as any observed background noise (potentially owing to nonspecific binding) would showcase itself as a false positive in this format. Each biotinylated *Bicycle* molecule was thus employed as a capture element (to be immobilized onto the polystreptavidin test line when wicked up the nitrocellulose membrane), and monoclonal IgGs utilized as the detection element were adsorbed onto platinum-based nanozyme probes. A clinically relevant concentration of SARS-CoV-2 N protein (1 ng·mL^–1^)^18^ was used as target antigen to evaluate the LFIA performance of these pairs through analysis of the test line intensity generated, and the absence of a visible test line when utilizing a subsequent blank sample.

As shown in Figure 3A, several pair combinations were successful in detecting 1 ng·mL^–1^ of SARS-CoV-2 N protein in the signal-amplified LFIA format. In particular, utilization of **B001** or **B002** as the capture agent in combination with **40143-MM05** or **40588-MM123** as the detection agent provided the greatest test line intensity at 1 ng·mL^–1^ SARS-CoV-2 N protein above any background signal indicated compared to a blank sample. It is interesting to note that the detection IgG biorecognition element choice appears to be a reasonable indicator for nonspecific binding (NSB), for example, **40588-MM124** consistently produced noticeable test line intensities with blank samples, irrespective of the capture *Bicycle molecule*. This also appears to be the case for the use of **40588-MM123** as the detection agent, albeit with a more diminished effect and varied effect. Additionally, **40143-MM08** proved to be largely ineffective as a detection agent for the detection of SARS-CoV-2 N protein in this format.

**Figure 3:**
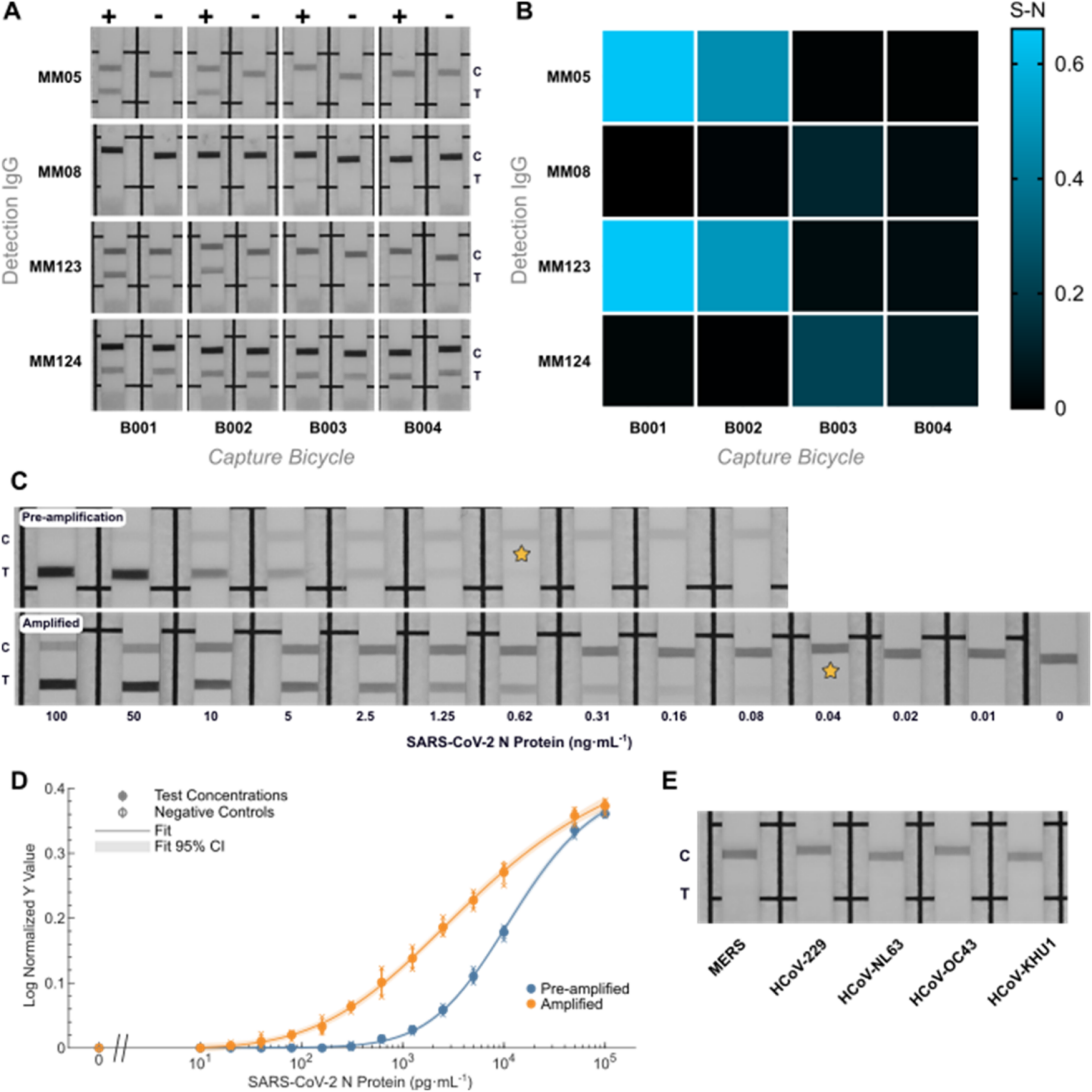
(A) Strips showcasing biorecognition element pair LFIA signal-minus-noise matrix at 1 ng·mL^–1^ SARS-CoV-2 N protein. (B) Corresponding biorecognition element pair LFIA signal-minus-noise matrix heatmap at 1 ng·mL^–1^ SARS-CoV-2 N protein. (C) Representative strips showing preamplification (top) and amplified (bottom) test line intensities at a serial dilution of SARS-CoV-2 N protein. Stars indicate LFIA strip with the lowest antigen concentration that can be seen visually by the naked eye. (D) Corresponding calibration curves showing normalized test line intensities for the preamplified and amplified detection of SARS-CoV-2 N protein in LFIA, data shown as mean ± S.D, *n* = 3. (E) LFIA cross-reactivity study for 1 μg·mL^–1^ of HCoV N proteins.

provided the greatest test line intensity at 1 ng·mL^–1^ SARS-CoV-2 N protein above any background signal indicated compared to a blank sample. It is interesting to note that the detection IgG biorecognition element choice appears to be a reasonable indicator for nonspecific binding (NSB), for example, **40588-MM124** consistently produced noticeable test line intensities with blank samples, irrespective of the capture *Bicycle molecule*. This also appears to be the case for the use of **40588-MM123** as the detection agent, albeit with a more diminished effect and varied effect. Additionally, **40143-MM08** proved to be largely ineffective as a detection agent for the detection of SARS-CoV-2 N protein in this format.

Overall, the relationship between pair combination performance in the LFIA screening process appears to be somewhat capricious in relation to their performance for the NLISA screen. This indicates that biorecognition element performance in plate-based diagnostics does not indicate a translation of performance in paper-based diagnostics and suggests that biorecognition pair application for different bioassay platforms should thus be evaluated independently. Although the pair **B001–40143-MM123** provided the greatest difference between test line intensity at 1 ng·mL^–1^ SARS-CoV-2 N protein and blank sample test line signal (Figure 3B); ultimately, biorecognition pair **B001–40143-MM05** was chosen as the final pair owing that there was no test line visible with a blank sample, even upon signal amplification, indicating a notable absence of NSB, while also providing similar test line intensity difference. The biorecognition pair **B001–40143-MM05** was tested using serially diluted SARS-CoV-2 N protein (concentration range for recombinant antigen from 100 to 0.01 ng·mL^–1^, representative strips shown in Figure 3C, see Figures S16 and S17 for all dilution series pre- and postamplification), and the resulting test line intensities resulted in a dose–response relationship (Figure 3D). Upon chromogenic signal amplification, greater test line intensities were observed, and subsequently, lower quantities of antigen were able to be detected.

As expected, the test line intensity gradually decreased as the dilution factor increased, and there were no observable test lines in the absence of antigen. The vLoD (visual limit of detection) was evaluated as the lowest antigen concentration at which a test line could be visually observed. In the preamplification scenario, the SARS-CoV-2 N protein was successfully detected at 0.62 ng·mL^–1^ (13 pM), whereas upon chromogenic amplification, a vLoD of 0.04 ng·mL^–1^ (0.83 pM) was achieved. This represents a LoD that surpasses that of commercial rapid antigen detection tests and, based on correlations between N antigen concentration and SARS-CoV-2 genome equivalents, suggests that this would be approaching sensitivities offered by PCR assays.^18^ Finally, a cross-reactivity study screening 1 μg·mL^–1^ of the nucleocapsid protein of the other human coronaviruses showed no visible test line, even upon catalytic signal amplification, highlighting the specificity of the utilized biorecognition elements and the specificity of the bioassay (Figure 3E).

### AuNP LFIA

With the performance of the **B001–40143-MM05** biorecognition pair for the nanozyme LFIA optimized, we thought it desirable to showcase the use of this biorecognition pair in a more familiar industry-standard gold nanoparticle (AuNP) colorimetric LFIA. Detection agent **40143-MM05** IgG was thus adsorbed onto 40 nm AuNPs, and in half-dipstick LFIA format utilizing biotinylated *Bicycle molecule* **B001** as the capture agent, a vLoD of 0.8 ng·mL^–1^ (17 pM) was achieved (see Figure S18). This essentially matched the preamplification performance of the half-dipstick nanozyme LFIA (see Figure 3). With the use of AuNPs as the nanoparticle probe being the gold standard for use in current PoC LFIA devices, we sought to demonstrate the performance of the developed AuNP half-dipstick LFIA utilizing the **B001–40143-MM05** biorecognition pair and conduct the bioassay utilizing pooled saliva as a clinically relevant sample matrix. Utilizing a sample buffer of 1:1 human pooled saliva to running buffer (0.2% (wt/v) β-casein and 0.1% (v/v) Tween 20 in PBS), a comparative performance of the half-dipstick LFIA was achieved with a vLoD of 0.8 ng·mL^–1^ (17 pM, representative strips shown in Figure 4A, see Figure S19 for all dilution series). This shows that the performance of the *Bicycle molecule*-integrated AuNP LFIA was unaffected by the use of a clinically relevant sample matrix when compared to a typical antigen-spiked buffer. Cross-reactivity experiments utilizing 1 μg·mL^–1^ N protein of the various HCoVs showed no visible test lines (Figure 4B).

**Figure 4:**
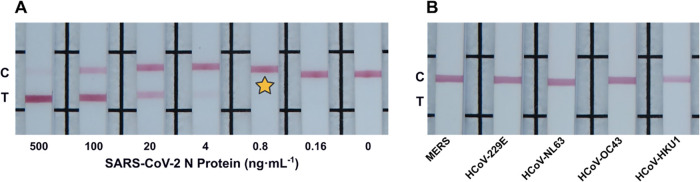
(A) Representative LFIA showing test line intensities at a serial dilution of SARS-CoV-2 N protein spiked in pooled saliva/running buffer matrix utilizing AuNPs. Star indicates LFIA strip with the lowest antigen concentration that can be seen visually by the naked eye. (B) LFIA cross-reactivity study for 1 μg·mL^–1^ of HCoV N proteins.

Notably, the limits of detection achieved here (0.8 ng·mL^–1^ for AuNP LFIA and 40 pg·mL^–1^ for nanozyme LFIA) are highly competitive when compared to state-of-the-art antibody-based sensors reported in the literature.^18^ Though it is worth noting that these results were obtained as a proof-of-concept study, direct comparison to commercial LFIAs is challenging given the extensive optimization and manufacturing controls applied to commercial products. Furthermore, precise benchmarking is complicated by the heterogeneity in validation metrics, as sensitivity is variably reported as protein concentration (pg/mL), viral genomic copies (copies/mL), or clinical cycle threshold (Ct), creating a challenge in directly correlating detection limits derived from recombinant proteins versus real-world viral loads.^18^ Nevertheless, the sensitivity demonstrated here highlights the utility of *Bicycles* as antibody alternatives.

## Conclusions

Constrained bicyclic peptides represent a novel class of biorecognition elements, and their minimalistic architecture comprises one of the smallest, if not the smallest, amino-acid-derived biorecognition moieties to be utilized in bioassays. We have demonstrated that they can serve as excellent complements to antibodies and can be effectively incorporated into plate- and paper-based diagnostics for protein antigen detection. Utilizing *Bicycle molecules* raised against the SARS-CoV-2 N protein NTD or CTD as capture reagents in *Bicycle*-IgG biorecognition element pairs, we achieved a limit of detection (LoD) of 250 pg·mL^–1^ in a standard sandwich ELISA format, and a visual LoD of 0.8 ng·mL^–1^ was attained in AuNP half-dipstick LFIA. Furthermore, by integrating catalytic platinum-based nanozymes, we further enhanced these detection limits, reaching an LoD of 97 pg·mL^–1^ in sandwich-format NLISA and an ultralow vLoD of 40 pg·mL^–1^ in signal-amplified, nanozyme-based, half-dipstick LFIA. In all the developed bioassays, no cross-reactivity was observed with respect to other HCoVs. We postulate that the minimal architecture of *Bicycle molecules* aids in minimizing off-target and nonspecific binding effects, while their synthetic production enables facile, site-selective modification that can prove extremely effective in the fabrication of diagnostic bioassays utilizing various nanomaterials as signal labels. *Bicycle molecules*, therefore, offer a viable alternative to antibodies in immunoassays, and the versatility of the platform to identify multiple differentiated hits supports the development of diverse bioassays for various disease targets.

## Materials and Methods

### Protein Production

All expression constructs of nucleocapsid domains were generated by SLIC cloning of PCR amplified codon-optimized DNA with overhangs compatible with *Bsa*I and *Hind*III linearized pExp-MBP plasmid (Addgene 112568) to create TEV cleavable His-MBP fusion proteins. All constructs were confirmed by dideoxy sequencing. Both the N-terminal domain (residues 47–173, Uniprot: P0DTC9) and the C-terminal domain (residues 247–368) of SARS-CoV-2 nucleocapsid proteins were produced without tags for crystallography and with a C-terminal Avi-tag for screening.

Protein expression was carried out in BL21­(DE3) cells in 2YT media. Expression was induced with 400 μM IPTG and carried on for 16 h at 18 °C. For Avi-tagged protein, the cells were cotransformed with a plasmid encoding for BirA biotin ligase under *araB* promoter for biotinylation during expression. BirA expression was induced before IPTG induction with 0.2% arabinose and 50 μM of biotin was added to the media. After expression, the cells were harvested by centrifugation and lysed with Emulsiflex C5 homogenizer (Avestin). Clarified lysate was applied to a 5 mL Ni-NTA column (Cube Biotech), and after washing, the protein was eluted with a buffer containing 250 mM imidazole. The eluate was then loaded onto a 5 mL amylose column (New England Biolabs) and fusion protein captured through its MBP moiety. After washing, the protein was eluted with 20 mM maltose. The eluate was treated with TEV protease (produced in-house) to remove the fusion partner, and the cleaved sample was passed through Ni-NTA agarose (Cube Biotech) column to capture the fusion partner, uncleaved fusion protein, and His-tagged TEV protease. The untagged protein was additionally purified by cation-exchange chromatography using a 5 mL HiTrap SP HP column (Cytiva). The N protein domains containing fractions were concentrated and purified further using size exclusion chromatography using Superdex 75 16/600 column (Cytiva) equilibrated in 20 mM Tris-acetate pH 7.2, 250 mM NaCl. Purified domains were concentrated to up to 20–25 mg/mL and flash frozen in small aliquots for storage at −80 °C.

### Identification of *Bicycle* Binders against SARS-CoV-2 Nucleocapsid Protein Using Phage Display

*Bicycle* bacteriophage (phage) libraries consist of linear peptides (10–20 amino acids in length) containing three cysteines which are cyclized in situ to form thioether-bonded bicyclic peptide libraries.^17^ These were used to pan for binders in selections against either full-length (ACROBiosystems, NUN-C81Q6), C-terminal domain, or N-terminal domain (as detailed above) recombinant proteins. Four rounds of selection were performed, using decreasing target concentrations of protein immobilized onto streptavidin magnetic beads, and binders were eluted at low pH. After round four, phage clones were isolated, Sanger sequenced, and tested for binding to proteins using *AlphaScreen* assay. Binders were synthesized as peptides using standard Fmoc solid-phase chemistry, and cyclization was performed as previously described.^32^ Affinity (*K*_d_, see Table S1) was determined using biolayer interferometry (BLI) on an Octed RED96e instrument against the protein constructs used in selections. BLI was selected as a label-free optical analytical technique that provides real-time kinetic data equivalent to Surface Plasmon Resonance (SPR), allowing for precise characterization of the interaction between immobilized protein targets and *Bicycle* binders. Representative N- and C-terminal protein binders were chosen to take further in the process, as synthesized with a PEG-linked biotin tag for further characterization purposes, using standard Fmoc chemistry.

### Crystallography

Co-crystals of both CTD constructs with BCY00018176 and NTD constructs with BCY00017628 were generated by screening the protein at 22–23 mg/mL in 20 mM Tris-acetate pH 7.2, 200 mM NaCl, with BCY00018176 and BCY00017628 in the crystallized samples at 3.1 mM and 2.0 mM, respectively, using LMB, BCS, Wizard I&II, and JCSG+ screens (Molecular Dimensions). Drops were set up using the Mosquito robotics system (SPT Labtech) with 0.2 μL of the protein:peptide complex solution and 0.2 μL of the screen solution using the sitting-drop vapor-diffusion method. Crystals of CTD: BCY00018176 complex were observed in 30% (w/v) 550M_20K 0.1 M MB2 pH 7.5, 10% MAA, while crystals of NTD:BCY00017628 were observed in 0.1 M HEPES pH 7 and 3.2 M (NH_4_)_2_SO_4_. The crystals were cryocooled in liquid nitrogen in the same solution for data collection. X-ray diffraction data were collected at Diamond Light Source synchrotron radiation sources and then processed using the pipedream package by Global Phasing Ltd.; structures were solved using Phaser^27^ from the CCP4 package.^28^ Models were iteratively refined and rebuilt by using Refmac^29^ and Coot^30^ programs. Ligand coordinates and restraints were generated from their SMILES strings using the AceDRG^31^ software from the CCP4 package.

The structures have been deposited in the Protein Data Bank under accession codes 9RXL (CTD in complex with BCY00018176) and 9S3N (NTD in complex with BCY00017628).

### General

All buffers were prepared using a Milli-Q d.d. H_2_O, and filtered through a 0.2 μm filter before use. Buffer pH was adjusted using 1 M HCl and 1 M NaOH, and the pH was tested each time before use. Gold nanoparticle and platinum nanocatalyst solutions were handled and stored in Protein LoBind sample tubes (Eppendorf) or glass sample vials.

### Buffer

The following buffers were used routinely: MES buffer (pH = 6.0, 100 mM); PBST (PBS + 0.05% (v/v) Tween 20); *blocking buffer* (PBS, 2% (wt/v) β-casein); *running buffer* (PBS + 0.2% (wt/v) β-casein + (v/v) 0.1% Tween 20).

### Synthesis of PtNC Colloid

Gold nanoparticle seeds with a diameter of ca. 15 nm were synthesized by the sodium citrate reduction of HAuCl_4_. In a typical synthesis, 2.5 mL of gold­(III) chloride trihydrate aqueous solution (10 mM, Sigma) was added to 47.2 mL of UltraPure distilled water under reflux at 100 °C. After 1 h, the reaction was initiated by fast injection of 0.3 mL of trisodium citrate dihydrate (0.5 M, Sigma) with vigorous stirring and refluxed for 15 min. The resulting ca. 15 nm gold nanoparticle (AuNP) seeds were cooled to room temperature and subsequently stored at 4 °C. Seed concentration was determined by UV–vis and initial gold concentration. 120 nm PtNCs were synthesized *via* reduction of chloroplatinic acid hydrate on gold seeds. In a typical synthesis, 620 μL (10 nM) of 15 nm Au seeds were mixed with 19.4 mL UltraPure distilled water, followed by the addition of 400 μL of 20% (wt/v) poly­(vinylpyrrolidone) (PVP MW 10 kDa, Sigma). The solution was vortexed briefly and incubated for 1 min for the polymer to coat and stabilize the particles. l-Ascorbic acid (800 μL, 100 mg·mL^–1^, Sigma) was then added to the mixture, followed by 800 μL of chloroplatinic acid hydrate (100 mM, Sigma), mixed, and immediately incubated at 65 °C for 45 min until the color of the solution changed from red to black, indicating successful deposition of platinum. PtNCs were then cooled to room temperature in a water bath, and excess reagents were removed through four sequential washing cycles at 1250 rcf for 12 min with resuspension into ultrapure distilled water.

### Preparation of PtNC Antibody Conjugate

To a Protein LoBind sample tube (Eppendorf), add 100 μL of PtNC (300 pM) and 2.25 μL of detection Ab (Sino Biological; 1 mg·mL^–1^) in a total of 10 μL of conjugation buffer (100 mM MES, pH 6). The mixture was incubated for 3 h at room temperature for antibody physisorption. Modified particles were subsequently blocked by the addition of 100 μL of blocking solution: 2% (wt/v) β-casein in PBS for 1 h at room temperature. Excess reagents were removed through three wash steps. Each wash step involves centrifugation at 1250 rcf for 12 min to pellet the conjugate, followed by the removal of the supernatant and addition of 200 μL of *running buffer*. After the final wash, the conjugate is resuspended in *running buffer* and stored at 4 °C.

### Preparation of AuNP Antibody Conjugate

To a glass vial was added 250 μL AuNP (citrate-capped, 40 nm, OD@530 = 1.0, BBI) and 1.50 μL of detection Ab (Sino Biological; cat: **41043-MM05**, 1 mg·mL^–1^) in a total of 50 μL conjugation buffer (100 mM MES, pH 6). The mixture was incubated for 3 h at room temperature for antibody physisorption. Modified particles were subsequently blocked by the addition of 25 μL of blocking solution: 2% (wt/v) β-casein in carbonate buffer (20 mM, pH 9.8) for 1 h at room temperature. The conjugate mixture was then transferred to a protein LoBind sample tube (Eppendorf), and excess reagents were removed through three wash steps. Each wash step involves centrifugation at 5000 rcf for 10 min to pellet the conjugate, followed by removal of the supernatant and addition of 500 μL wash buffer (20 mM MES, pH 6 + 0.05% (v/v) Tween 20). After the final wash, the conjugate is resuspended in wash buffer at OD@530 = 1.0 and stored at 4 °C.

### Dynamic Light Scattering

DLS measurements were performed on a Zetasizer Nano ZS instrument (Malvern) equipped with a 633 nm laser. Measurement parameters were optimized by Zetasizer Nano software v8.02, and samples were equilibrated to 21 °C before measurements. Values for the size vs intensity, intensity diameter, *Z*-average diameter, and polydispersity index were calculated by the software and exported without further manipulation. Changes in diameter were calculated as the difference in the *Z*-average diameter between the product and the starting material.

### TEM

Sample preparation for TEM characterization was undertaken by diluting PtNCs and 15 nm AuNPs to a final concentration of 30 pM and 5 nM, respectively, in ultrapure distilled water. 2 μL of the sample was then drop-cast onto a carbon-coated square mesh copper grid (CF-300-Cu, Electron Microscopy Sciences) and left to dry in air overnight. The images were then taken using a JEOL 2100F set at 200 kV with a beam current of 101 μA, and micrographs were obtained using a Gatan Orius SC1000 camera.

### UV–Vis Spectroscopy

UV–vis were obtained on a Nanodrop 2000c (Thermo Scientific) running in cuvette mode, scanning 200–840 nm in 1 nm steps. Baseline correction was achieved by running a blank containing only the sample buffer and automatically subtracted from the data by the software. Samples were measured in microcuvettes with a path length of 1 cm.

### Heatmap ELISA

To appropriate wells of a Thermo Scientific, Pierce Streptavidin Coated High Capacity Plate (Clear, 96-Well) was added 100 μL of biotinylated *Bicycle molecule* (**B001**, **B002**, **B003**, or **B004**) in PBS at 1 μM and incubated for 2 h at room temperature. The plate was then washed (3×) with PBST (400 μL·well^–1^) and 100 μL recombinant SARS-CoV-2 nucleocapsid protein (Sino Biological; cat: 40588-V08B) in PBST at 50 ng·mL^–1^ was added and incubated at room temperature for 30 min with PBST as a negative control. The wells were washed again (3×) with PBST and 100 μL of detection IgG (Sino Biological; cat: **41043-MM05**, **41043-MM08**, **40588-MM123**, or **40588-MM124**) in PBST at 0.25 ng·mL^–1^ was added to appropriate wells and incubated at room temperature for 30 min. The wells were washed again (3×) with PBST, and 100 μL of anti-mouse HRP (Abcam; cat: ab97046) in PBST (1:40,000) was added to each well and incubated at room temperature for 30 min. The wells were washed again (3×) with PBST before 100 μL of a freshly prepared TMB solution (1.6% (v/v) TMB (6 mg·mL^–1^) in DMSO + 0.4% (v/v) 1% H_2_O_2_ in 40 mM citrate buffer, pH 5.5) was added to each well, after which the plate was shielded from light for 30 min. H_2_SO_4_ (4 M, 50 μL·well^–1^) was added to each well, at which point the OD at 450 nm was recorded by SpectraMax M5 microplate reader (Molecular Devices) and analyzed using SoftMax Pro 7.1.2. All assays were run in triplicate, and the data were analyzed using GraphPad Prism v.10.1.0.

### Serial Dilution ELISA

To appropriate wells of a Thermo Scientific, Pierce Streptavidin Coated High Capacity Plate (Clear, 96-Well) was added 100 μL of biotinylated *Bicycle* molecule (**B001**, **B002**, **B003**, or **B004**) in PBS at 1 μM and incubated for 2 h at room temperature. The plate was then washed (3×) with PBST, and 100 μL of a serial dilution of recombinant SARS-CoV-2 nucleocapsid protein (Sino Biological; cat: 40588-V08B) in PBST was added and incubated at room temperature for 30 min with PBST as a negative control. The wells were washed again (3×) with PBST and 100 μL detection IgG (Sino Biological; cat: **41043-MM05**, **41043-MM08**, **40588-MM123** or **40588-MM124**) in PBST at 0.25 ng·mL^–1^ was added to appropriate wells and incubated at room temperature for 30 min. The wells were washed again (3×) with PBST, and 100 μL of anti-mouse HRP (Abcam; cat: ab97046) in PBS (1:40,000) was added to each well and incubated at room temperature for 30 min. The wells were washed again (3×) with PBST before 100 μL of a freshly prepared TMB solution (1.6% (v/v) TMB (6 mg·mL^–1^) in DMSO + 0.4% (v/v) 1% H_2_O_2_ in 40 mM citrate buffer, pH 5.5) was added to each well, after which the plate was shielded from light for 30 min. H_2_SO_4_ (4 M, 50 μL·well^–1^) was added to each well, at which point the OD at 450 nm was recorded by SpectraMax M5 microplate reader (Molecular Devices) and analyzed using SoftMax Pro 7.1.2. All assays were run in triplicate, and the data were analyzed using Detection Limit Fitting Tool v1.0.0.0, Matlab 2021a (https://github.com/bensmiller/detection-limit-fitting).^21^

### Heatmap NLISA

To appropriate wells of a Thermo Scientific, Pierce Streptavidin Coated High Capacity Plate (Clear, 96-Well) was added 100 μL of biotinylated *Bicycle molecule* (**B001**, **B002**, **B003**, or **B004**) in PBS at 1 μM and incubated for 2 h at room temperature. The plate was then washed (3×) with PBST, and 100 μL of recombinant SARS-CoV-2 nucleocapsid protein (Sino Biological; cat: 40588-V08B) in PBST at 10 ng·mL^–1^ was added and incubated at room temperature for 30 min with PBST as a negative control. The wells were washed again (3×) with PBST and 100 μL of PtNC-conjugated IgG (Sino Biological; cat: **41043-MM05**, **41043-MM08**, **40588-MM123** or **40588-MM124**) in PBST at 2 pM was added to appropriate wells and incubated at room temperature for 30 min. The wells were washed again (3×) with PBST before 100 μL of a freshly prepared TMB solution (1.6% (v/v) TMB (6 mg·mL^–1^) in DMSO + 0.4% (v/v) 5% H_2_O_2_ in 50 mM citrate buffer, pH 5.0) was added to each well, after which the plate was shielded from light for 30 min. H_2_SO_4_ (4 M, 50 μL·well^–1^) was added to each well, at which point the OD at 450 nm was recorded by SpectraMax M5 microplate reader (Molecular Devices) and analyzed using SoftMax Pro 7.1.2. All assays were run in triplicate, and the data were analyzed using GraphPad Prism v.10.1.0.

### Serial Dilution NLISA

To appropriate wells of a Thermo Scientific, Pierce Streptavidin Coated High Capacity Plate (Clear, 96-Well) was added 100 μL of biotinylated *Bicycle molecule* (**B001**, **B002**, **B003**, or **B004**) in PBS at 1 μg·mL^–1^ and incubated for 2 h at room temperature. The plate was then washed (3×) with PBST, and 100 μL of a serial dilution of recombinant SARS-CoV-2 nucleocapsid protein (Sino Biological; cat: 40588-V08B) in PBST was added and incubated at room temperature for 30 min with PBST as a negative control. The wells were washed again (3×) with PBST, and 100 μL of PtNC-conjugated IgG (Sino Biological; cat: **41043-MM05**, **41043-MM08**, **40588-MM123**, or **40588-MM124**) in PBST at 2 pM was added to appropriate wells and incubated at room temperature for 30 min. The wells were washed again (3×) with PBST before 100 μL of a freshly prepared TMB solution (1.6% (v/v) TMB (6 mg·mL^–1^) in DMSO + 0.4% (v/v) 5% H_2_O_2_ in 50 mM citrate buffer, pH 5.0) was added to each well, after which the plate was shielded from light for 30 min. H_2_SO_4_ (4 M, 50 μL·well^–1^) was added to each well, at which point the OD at 450 nm was recorded by SpectraMax M5 microplate reader (Molecular Devices) and analyzed using SoftMax Pro 7.1.2. All assays were run in triplicate, and the data were analyzed using Detection Limit Fitting Tool v1.0.0.0, Matlab 2021a (https://github.com/bensmiller/detection-limit-fitting).^21^

### PtNC Lateral Flow Immunoassays

All PtNC lateral flow assays for SARS-CoV-2 N protein detection were performed by submerging a Mologic Ltd. polystreptavidin-printed strip into a Corning 96-well Clear Flat Bottom Polystyrene NBS Microplate containing the following solution in each well: 10 μL of biotinylated *Bicycle* molecule (1 μM), 50 μL of SARS-CoV-2 N protein spiked into *running Buffer*, and 15 μL of PtNC antibody conjugate (100 pM). When the solution had fully wicked up the strip (ca. 10 min), the strip was moved into another well filled with 100 μL of *running buffer* for 5 min. Next, the strip was immersed in another well for 5 min filled with 400 μL of freshly prepared PtNC development solution containing a modified Pierce CN/DAB (4-chloro-1-naphthol/3,3′-diaminobenzidine, tetrahydrochloride) Substrate Kit (Thermo Scientific) adjusted with hydrogen peroxide solution 30% (w/w) (Sigma) to a final added peroxide concentration of 4 M. Finally, the strip was moved into a well containing 400 μL of purified water for 1 min to stop the reaction. Strips were imaged with a CanonPowerShot G15 camera 5 min after removal from the water. Test line densitometry was achieved using ImageJ. Briefly, the raw images were imported into ImageJ and converted to gray scale. A region of interest (ROI) was drawn around the test line, and the pixel density was counted using the software. To control for lighting differences across the image, an identical ROI was drawn around the printed grid line directly below each strip, and the pixel density from the test lines was normalized to these values.

### AuNP Lateral Flow Immunoassays

All AuNP lateral flow assays for SARS-CoV-2 N protein detection were performed by submerging a Mologic Ltd. polystreptavidin-printed strip into a Corning 96-well Clear Flat Bottom Polystyrene NBS Microplate containing the following solution in each well: 10 μL of biotinylated *Bicycle* molecule (**B001**, 1 μM), 50 μL of SARS-CoV-2 N protein spiked into either FBST or 1:1 spiked saliva *Running Buffer*, and 20 μL of AuNP antibody conjugate (OD@530 = 1.0). When the solution had fully wicked up the strip (ca. 10 min), the strip was allowed to dry for 5 min and imaged with an iPhone 13 or CanonPowerShot G15 camera. Test line densitometry was achieved using ImageJ. Briefly, the raw images were imported into ImageJ and converted to gray scale. A region of interest (ROI) was drawn around the test line, and the pixel density was counted using the software. To control for lighting differences across the image, an identical ROI was drawn around the printed grid line directly below each strip, and the pixel density from the test lines was normalized to these values.

## Supplementary Material



## Notes

Validation data underlying this work, including target sequences, bicyclic peptide sequences and binding affinities with corresponding analytical HPLC characterization, structural data for peptide–nucleocapsid complexes, and full assay performance metrics; reports for ELISA, NLISA, and LFIA calibration data, limits of detection, statistical analyses, and nanoparticle synthesis and conjugation characterization, providing supporting evidence for the results discussed in the main text (PDF)

## Abbreviations

NTD: N-terminal domain
CTD: C-terminal domain
SARS-CoV-2: severe acute respiratory syndrome coronavirus 2
ELISA: enzyme-linked immunosorbent assay
NLISA: nanozyme-linked immunosorbent assay
PoC: point-of-care
LFIA: lateral flow immunoassay
IgG: immunoglobulin G
HCoV: human coronavirus
MERS: Middle East respiratory syndrome
LoD: limit of detection
PtNC: platinum nanocatalyst
DLS: dynamic light scattering
TEM: transmission electron microscopy
AuNP: gold nanoparticle
vLoD: visual limit of detection
HRP: horseradish peroxidase
TMB: 3,3′,5,5′-tetramethylbenzidine
