# Supplementary material for: Utilizing Constrained Bicyclic Peptides for In Vitro Diagnostics
Source: ACS Nano. 2026 Feb 13;20(7):5928–39. doi: 10.1021/acsnano.5c19041 (PMC12947737; doi:10.1021/acsnano.5c19041)
Supplement: Supplementary file 1 [file nn5c19041_si_001.pdf]

Supplementary Information

Utilizing Constrained Bicyclic Peptides for in vitro Diagnostics

André Shamsabadi†, Adam Creamer†, Christy J. Sadler†, Aida Abdelwahed†, Katherine U. Gaynor,‡ Yuliya Demydchuk,‡ Gabriela Ivanova-Berndt,‡ Katerine Van Rietschoten,‡ Paul Beswick,‡ Liuhong Chen,‡ Gustavo Arruda Bezerra,‡ Aleksei Lulla,† Paul Brear,† Marko Hyvönen,† Michael J. Skynner,‡ Molly M. Stevens†*

†Department of Materials, Department of Bioengineering and Institute of Biomedical Engineering, Imperial College London, London, SW7 2AZ, U.K.

‡Bicycle Therapeutics, Portway Building, Granta Park, Cambridge, CB21 6GS, U.K.

†Department of Biochemistry, University of Cambridge, Cambridge CB2 1GA, U.K.

Text S1: Protein Sequences

>SARS-CoV-2 nucleoprotein C-terminal domain
GSTKKSAAEASKKPRQKRTATKAYNVTQAFGRRGPEQTQGNFGDQELIRQGTDYKHWPQIAQFAPSASAFFGMSRIGMEVTPSGT
WLTYTGAIKLDDKDPNFKDQVILLNKHIDAYKTFP

>SARS-CoV-2 nucleoprotein C-terminal domain with a C-terminal Avi-tag
GSTKKSAAEASKKPRQKRTATKAYNVTQAFGRRGPEQTQGNFGDQELIRQGTDYKHWPQIAQFAPSASAFFGMSRIGMEVTPSGT
WLTYTGAIKLDDKDPNFKDQVILLNKHIDAYKTFP GSSGLNDIFEAQKIEWHEA

>SARS-CoV-2 nucleocapsid N-terminal domain
GSNNTASWFTALTQHGKEDLKFPRGQGVPINTNSSPDDQIGYYRRATRRIRGGDGKMKDLSPRWYFYYLGTGPEAGLPYGANKDGII
WVATEGALNTPKDHIGTRNPANNAAIVLQLPQGTTLPKGFYA

>SARS-CoV-2 nucleocapsid N-terminal domain with a C-terminal Avi-tag
GSNNTASWFTALTQHGKEDLKFPRGQGVPINTNSSPDDQIGYYRRATRRIRGGDGKMKDLSPRWYFYYLGTGPEAGLPYGANKDGII
WVATEGALNTPKDHIGTRNPANNAAIVLQLPQGTTLPKGFYASSGLNDIFEAQKIEWHEA

Data for Bicyclic Peptides

Table S1: Binding affinities of peptides to N Protein using BLI.

Bicyclic Peptide (biotinylated)	Scaffold	Peptide Sequence																	MW	GeoMean K_d (nM) of naked format peptide
B001	TCMT	A	C_{i}	T	T	D	F	C_{ii}	N	N	R	Y	V	V	N	R	C_{iii}	A	3685.3	18.2*
B002	TCMT	A	C_{i}	W	T	D	F	C_{ii}	N	N	R	Y	V	V	N	R	C_{iii}	A	3685.3	25.3*
B003	TATA	A	C_{i}	S	T	D	V	C_{ii}	K	L	T	G	I	A	I	P	C_{iii}	A	2868.5	1536#
B004	TATA	A	C_{i}	M	Q	N	P	C_{ii}	R	W	V	N	I	P	C_{iii}	A			2908.5	3869#

TATA = 1,1',1''-(1,3,5-triazinane-1,3,5-triyl)triprop-2-en-1-one (triacryloylhexahydro-s-triazine),
TCMT = 2,4,6-tris(chloromomethyl)-s-triazine

* Binding to N-terminal domain of N protein
# Binding to C-terminal domain of N protein

Full length (ACROBiosystems, NUN-C81Q6), C-terminal domain, or N-terminal domain (both Hyvönen lab) recombinant proteins were used to pan for binders in selections.

Trace S1: B001 analytical HPLC trace

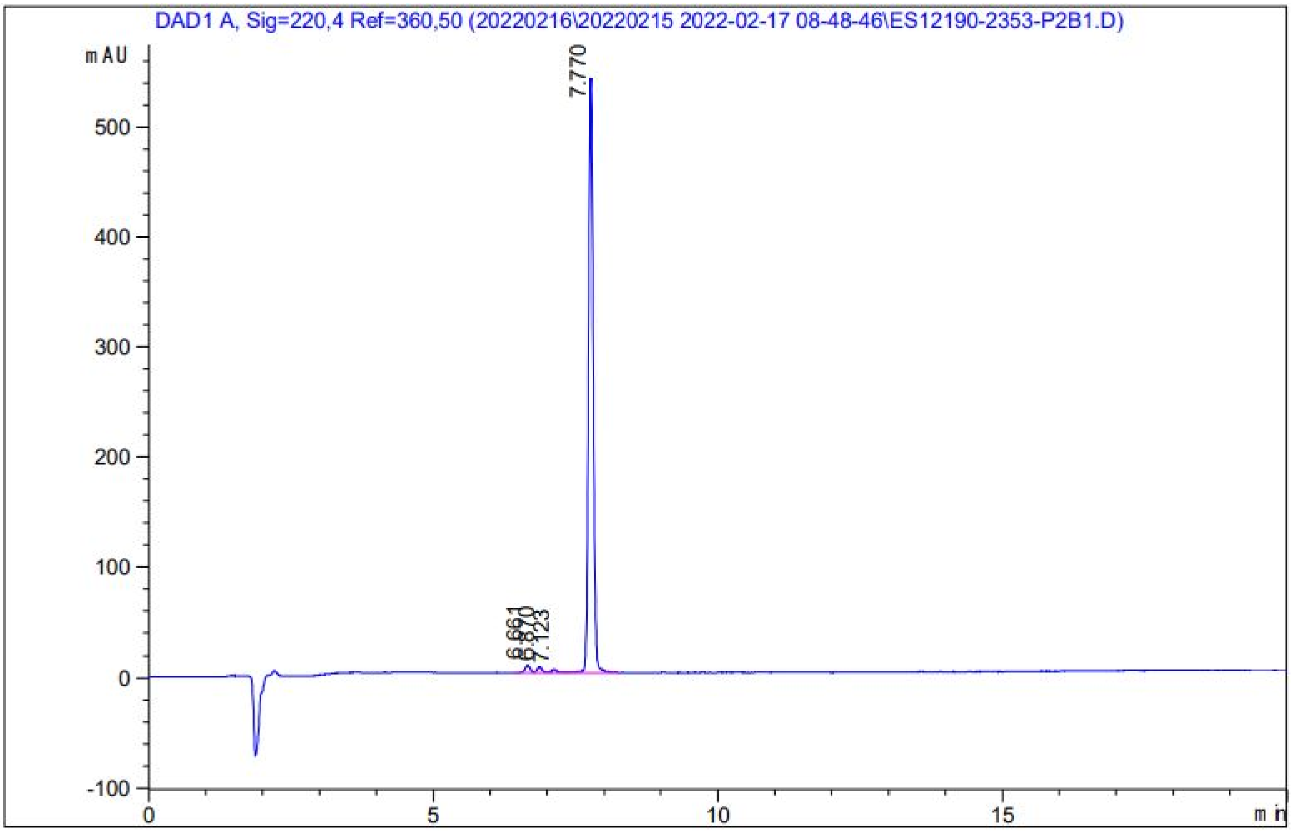

Parameters:

Mobile Phase	**A:** 0.1% TFA in H_2O **B:** 0.1% TFA in ACN
Flow Rate (mL/min)	1.0
Column	Gemini-NX C18 5 µm 110A 150*4.6 mm
Retention Time (min)	7.77

Trace S2: B002 analytical HPLC trace

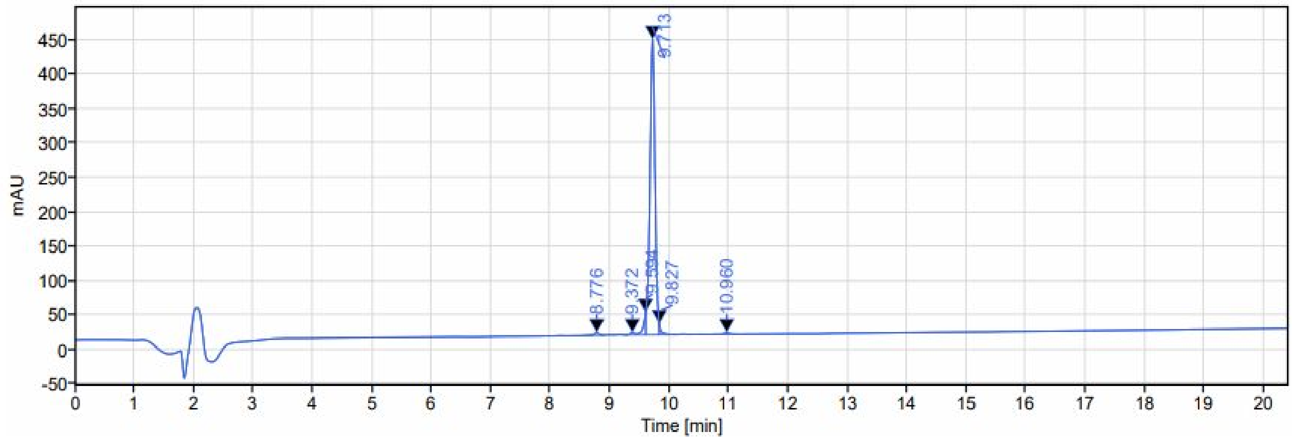

Parameters:

Mobile Phase	**A:** 0.1% TFA in H_2O **B:** 0.1% TFA in ACN
Flow Rate (mL/min)	1.0
Column	Gemini-NX C18 5µm 110A 150*4.6 mm
Retention Time (min)	9.71

Trace S3: B003 analytical HPLC trace

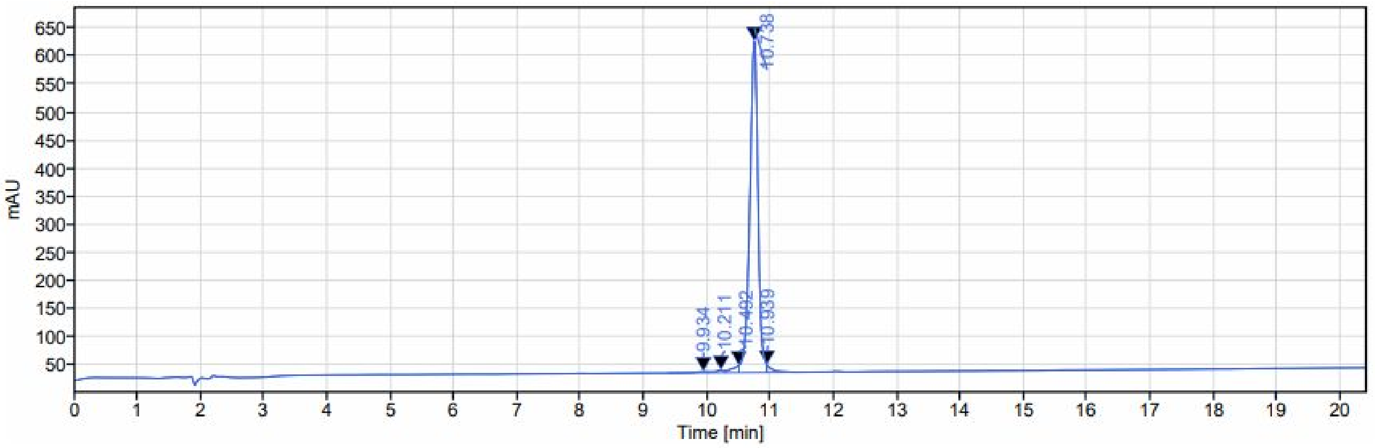

Mobile Phase	**A:** 0.1% TFA in H_2O **B:** 0.1% TFA in ACN
Flow Rate (mL/min)	1.0
Column	Gemini-NX C18 5µm 110A 150*4.6 mm
Retention Time (min)	10.74

Trace S4: B004 analytical HPLC trace

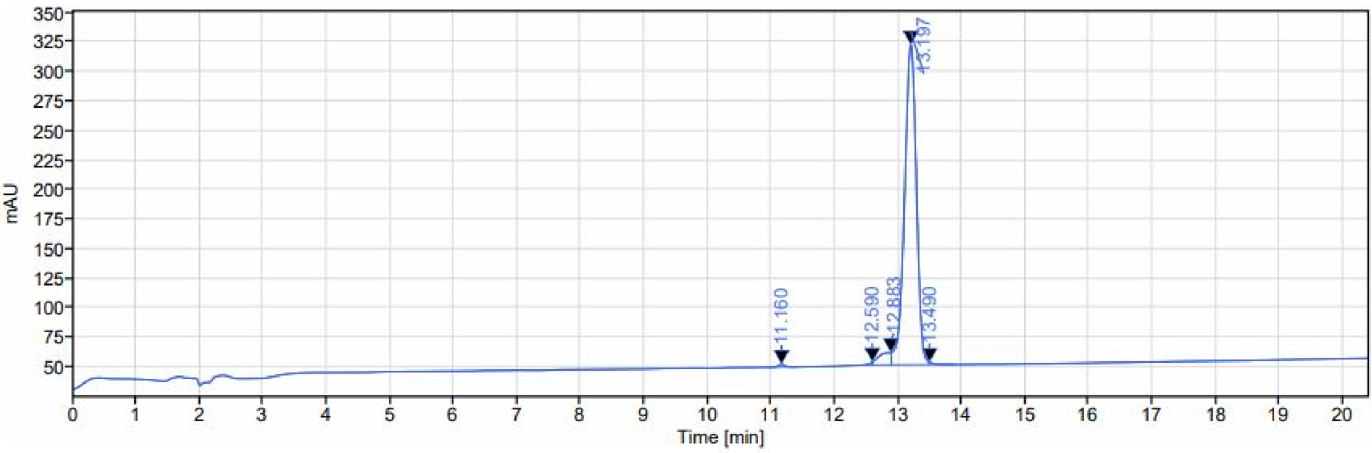

Mobile Phase	**A:** 0.1% TFA in H_2O **B:** 0.1% TFA in ACN
Flow Rate (mL/min)	1.0
Column	Gemini-NX C18 5µm 110A 150*4.6 mm
Retention Time (min)	13.49

ELISA Heatmap SD

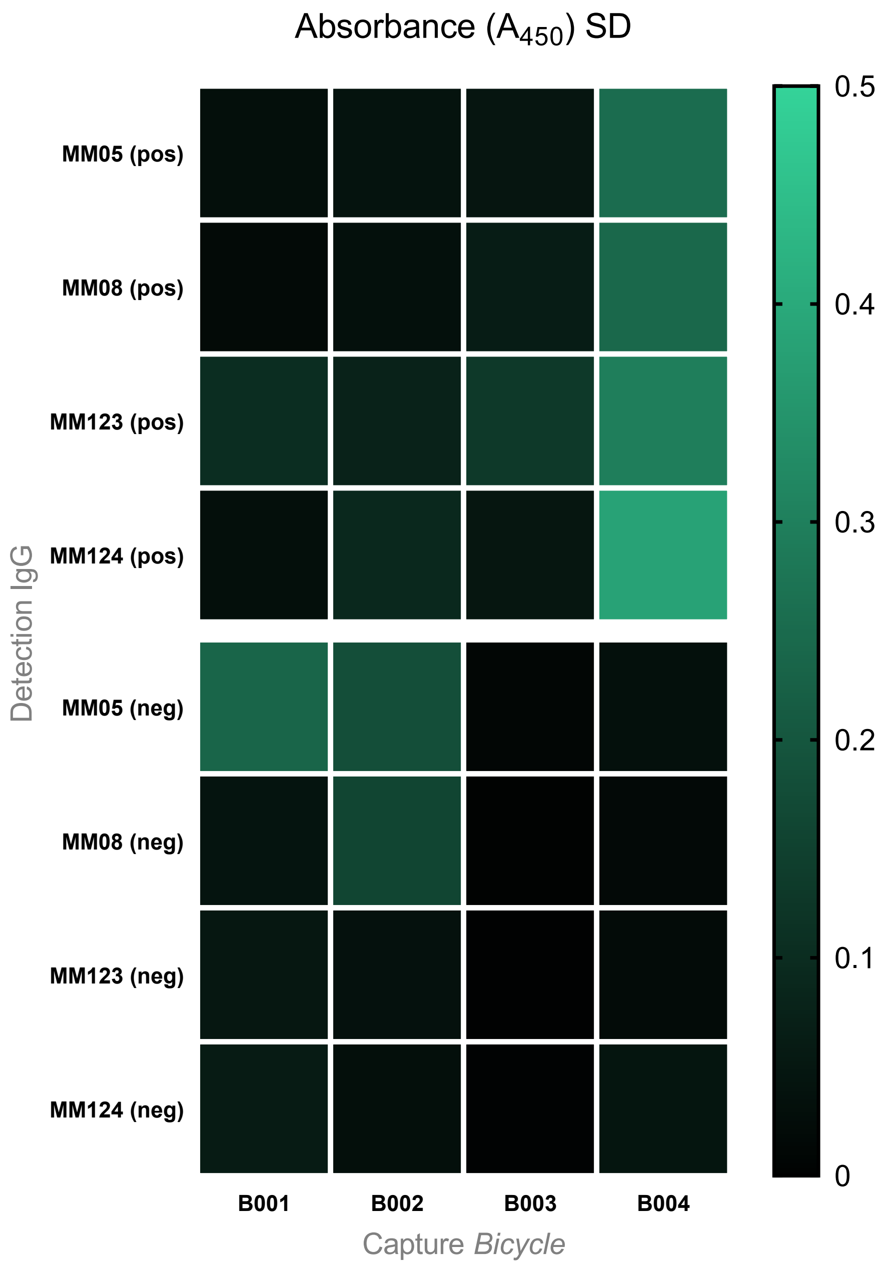

Figure S1: ELISA biorecognition pair standard deviation heatmap, n = 3.

ELISA Heatmap CV

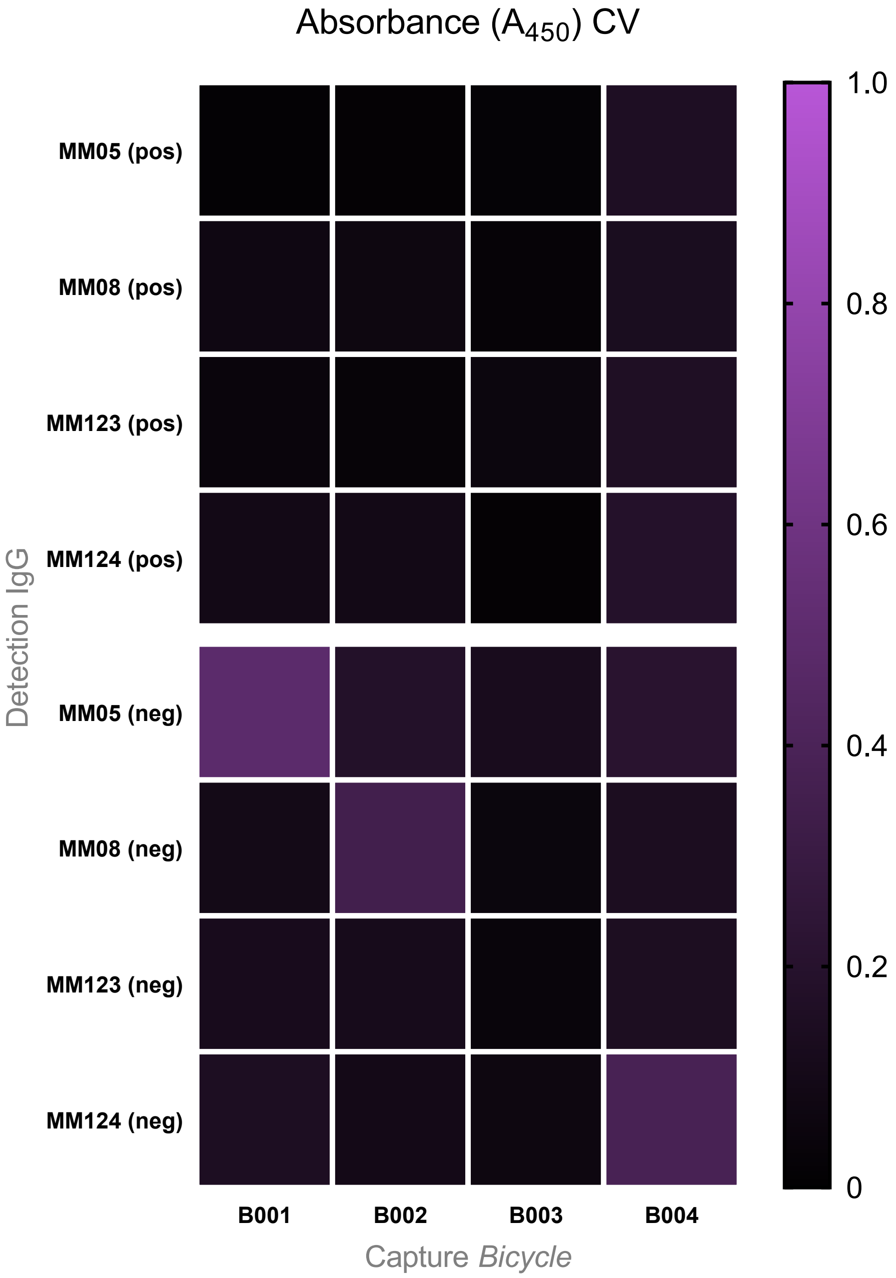

Figure S2: ELISA biorecognition pair standard deviation coefficient of variation, n = 3.

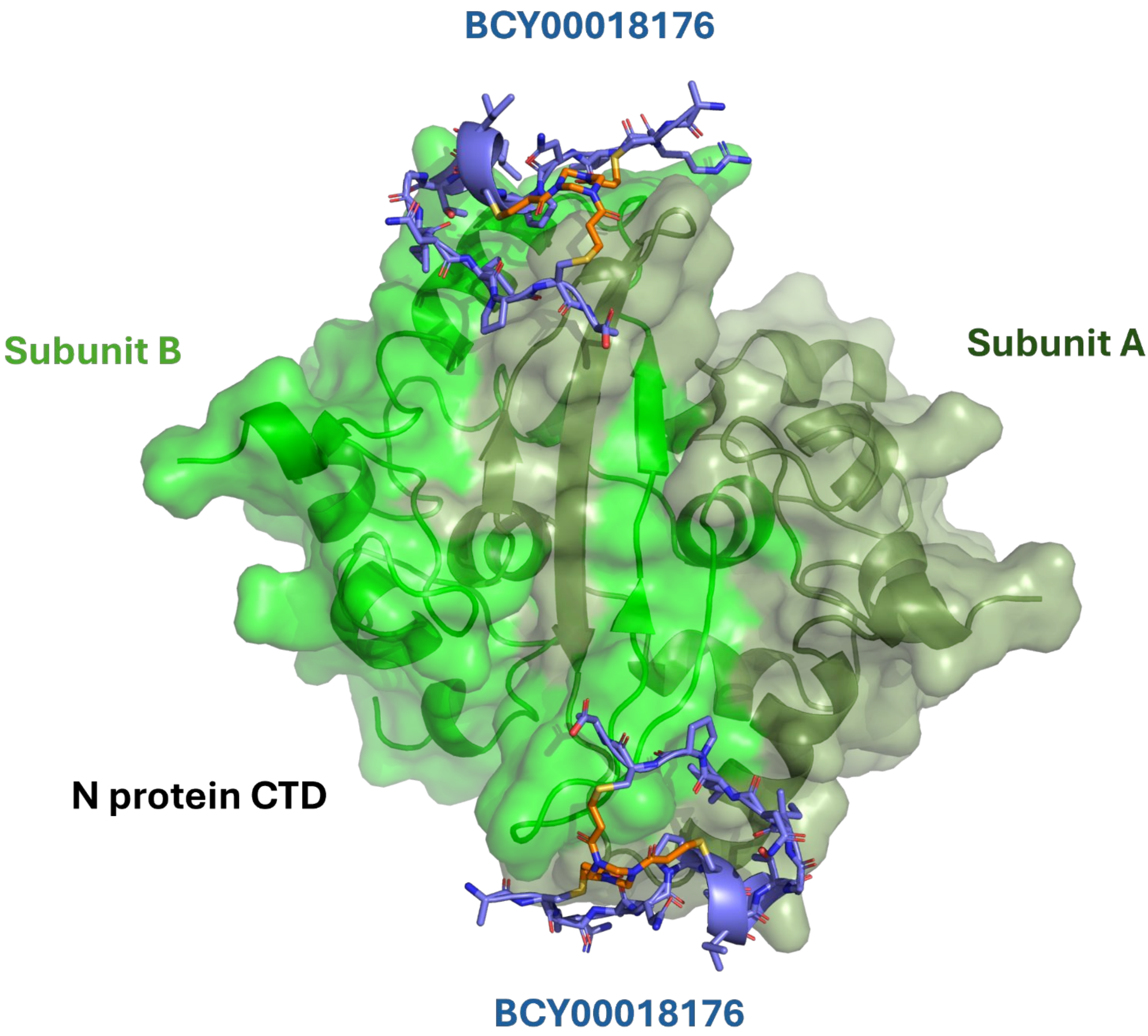

PDB code	9RXL
Ligand	**BCY00018176**
Protein	Nucleocapsid CTD
Data Collection:	
Beamline	Diamond Light Source, I04
Wavelength (Å)	0.9795
Resolution range (Å)	61.94 - 1.46 (1.54 - 1.46)
Space group	P 4_3 2_1 2
Cell (a b c)	73.04 73.04 116.86
Cell (α β γ) (°)	90.00 90.00 90.00
Total reflections	3616304 (522583)
Unique reflections	55845 (8002)
Multiplicity	64.8 (65.3)

Completeness (%)	100.0 (100.0)
Mean I/sigma(I)	18.6 (0.7)
R-merge	0.206 (9.66)
R-pim	0.026 (1.20)
CC-half	1.000 (0.42)
Refinement:	
Refinement resolution range (Å)	61.94 - 1.46 (1.50 - 1.46)
No. reflections	52919 (8002)
No. reflections (Rfree)	2705 (214)
R-factor	0.183 (0.373)
Rfree	0.214 (0.378)
Number of all atoms	2519
Number of protein atoms	1880
Number of ligand atoms	276
Number of solvent atoms	363
Average B-factor (Å^2)	25.8
RMS(bonds) (Å)	0.013
RMS(bond angles) (°)	1.81
RMS(dihedral angles) (°)	7.071

Figure S3: Surface and cartoon representation of N protein CTD. Subunit A is shown in dark green, while subunit B is shown in green. BCY00018176 is shown as blue sticks with the scaffold as orange sticks.

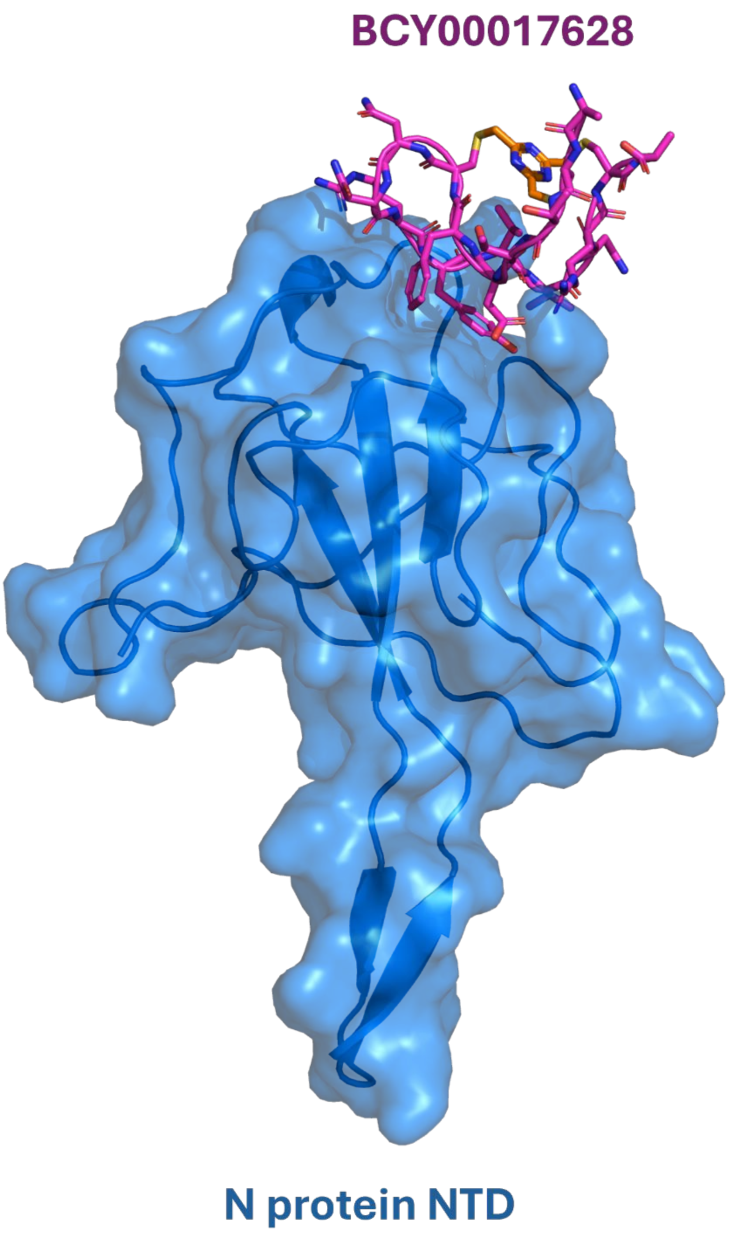

PDB code	9S3N
Ligand	**BCY00017628**
Protein	Nucleocapsid NTD
Data Collection:	
Beamline	Diamond Light Source, I04-1
Wavelength (Å)	0.9999
Resolution range (Å)	65.01 - 1.93 (1.96 - 1.93)
Space group	P 4_3 2_1 2
Cell (a b c)	91.94 91.94 68.56
Cell (α β γ) (°)	90.00 90.00 90.00
Total reflections	568153 (23054)
Unique reflections	22732 (1100)
Multiplicity	25.0 (21.0)

Completeness (%)	100.0 (98.6)
Mean I/sigma(I)	13.9 (0.4)
R-merge	0.132 (5.97)
R-pim	0.027 (1.33)
CC-half	0.996 (0.33)
Refinement:	
Refinement resolution range (Å)	45.97 - 1.93 (2.02 - 1.93)
No. reflections	22506 (2488)
No. reflections (Rfree)	1084 (121)
R-factor	0.247 (0.351)
Rfree	0.262 (0.376)
Number of all atoms	1203
Number of protein atoms	1115
Number of ligand atoms	148
Number of solvent atoms	79
Average B-factor ($Å^2$)	56.77
RMS(bonds) (Å)	0.007
RMS(bond angles) (°)	0.781
RMS(dihedral angles) (°)	15.531

Figure S4: Surface and carton representation of N protein NTD in cyan. BCY00017628 is shown as magenta sticks with the scaffold in orange.

ELISA Pair Tabulated Parameters

Table S2: Capture B003, Detection 40588-MM124 Detection Limit Fitting outputs.

Capture B003, Detection 40588-MM124 Detection Limit Fitting Outputs (5 parameter model)

Parameter	Value
**LOD**	253.8982
**LOD lower**	169.317
**LOD upper**	380.2381
**Confidence level negatives**	0.05
**Confidence level positives**	0.05
**Confidence level variances**	0.05
**L_D**	0.0018244
**L_C**	0.0086553
**Units**	pg/mL

Table S3: Capture B003, Detection 40588-MM124 sigmoidal regression curve parameters. The data have been extracted from the four-parameter model used to fit the standard curve.

Capture B003, Detection 40588-MM124 Tabulated Parameters (4 parameter model)

Best-fit values	
**Bottom**	0.09553
**Hillslope**	1.426
**Top**	2.762
**EC50**	4.887
**R squared**	0.9991

Table S4: Capture B003, Detection 41043-MM08 Detection Limit Fitting outputs.

Capture B003, Detection 41043-MM08 Detection Limit Fitting Outputs (5 parameter model)

Parameter	Value
**LOD**	505.2173
**LOD lower**	381.5494
**LOD upper**	668.7595
**Confidence level negatives**	0.05
**Confidence level positives**	0.05
**Confidence level variances**	0.05
L_D	0.0071318
L_C	0.01887
**Units**	pg/mL

Table S5: Capture B003, Detection 41043-MM08 sigmoidal regression curve parameters. The data have been extracted from the four-parameter model used to fit the standard curve.

Capture B003, Detection 41043-MM08 Tabulated Parameters (4 parameter model)

Best-fit values	
**Bottom**	0.08664
**Hillslope**	1.402
**Top**	2.734
**EC50**	5.656
**R squared**	0.9979

Table S6: Capture B003, Detection 40588-MM123 Detection Limit Fitting Outputs.

Capture B003, Detection 40588-MM123 Detection Limit Fitting Outputs (5 parameter model)

Parameter	Value
**LOD**	606.3152
**LOD lower**	420.1011
**LOD upper**	874.6794
**Confidence level negatives**	0.05
**Confidence level positives**	0.05
**Confidence level variances**	0.05
**L_D**	0.0014776
**L_C**	0.011939
**Units**	pg/mL

Table S7: Capture B003, Detection 40588-MM123 sigmoidal regression curve parameters. The data have been extracted from the four-parameter model used to fit the standard curve.

Capture B003, Detection 40588-MM123 Tabulated Parameters (4 parameter model)

Best-fit values	
**Bottom**	0.07569
**Hillslope**	1.448
**Top**	2.715
**EC50**	6.983
**R squared**	0.9981

Table S8: Capture B003, Detection 40143-MM05 Detection Limit Fitting Outputs.

Capture B003, Detection 40143-MM05 Detection Limit Fitting Outputs (5 parameter model)

Parameter	Value
**LOD**	1680.1337
**LOD lower**	991.7809
**LOD upper**	2845.2814
**Confidence level negatives**	0.05
**Confidence level positives**	0.05
**Confidence level variances**	0.05
L_D	0.003742
L_C	0.046731
**Units**	pg/mL

Table S9: Capture B003, Detection 40143-MM05 sigmoidal regression curve parameters. The data have been extracted from the four-parameter model used to fit the standard curve.

Capture B003, Detection 40143-MM05 Tabulated Parameters (4 parameter model)

Best-fit values	
**Bottom**	0.08882
**Hillslope**	1.546
**Top**	2.528
**EC50**	7.757
**R squared**	0.9803

ELISA Best Pairs ANOVA Test

Table S10: ELISA Best biorecognition pair ANOVA test (Tukey-Kramer post-hoc test)

Comparison 1	Comparison 2	Lower CI of log[LOD (pg/mL)] difference	CI of log[LOD (pg/mL)] difference	Upper CI of log[LOD (pg/mL)] difference	P-value
BCY003, 40143-MM05	BCY003, 40143-MM08	1.89E-01	5.21E-01	8.53E-01	5.63E-04
BCY003, 40143-MM05	BCY003, 40588-MM123	1.10E-01	4.42E-01	7.74E-01	4.40E-03
BCY003, 40143-MM05	BCY003, 40588-MM124	4.86E-01	8.18E-01	1.15E+00	6.01E-08
BCY003, 40143-MM08	BCY003, 40588-MM123	-4.11E-01	-7.89E-02	2.53E-01	9.24E-01
BCY003, 40143-MM08	BCY003, 40588-MM124	-3.50E-02	2.97E-01	6.29E-01	9.58E-02
BCY003, 40588-MM123	BCY003, 40588-MM124	4.39E-02	3.76E-01	7.08E-01	2.02E-02

Capture B003, Detection 40588-MM124 Pair Calibration Curve

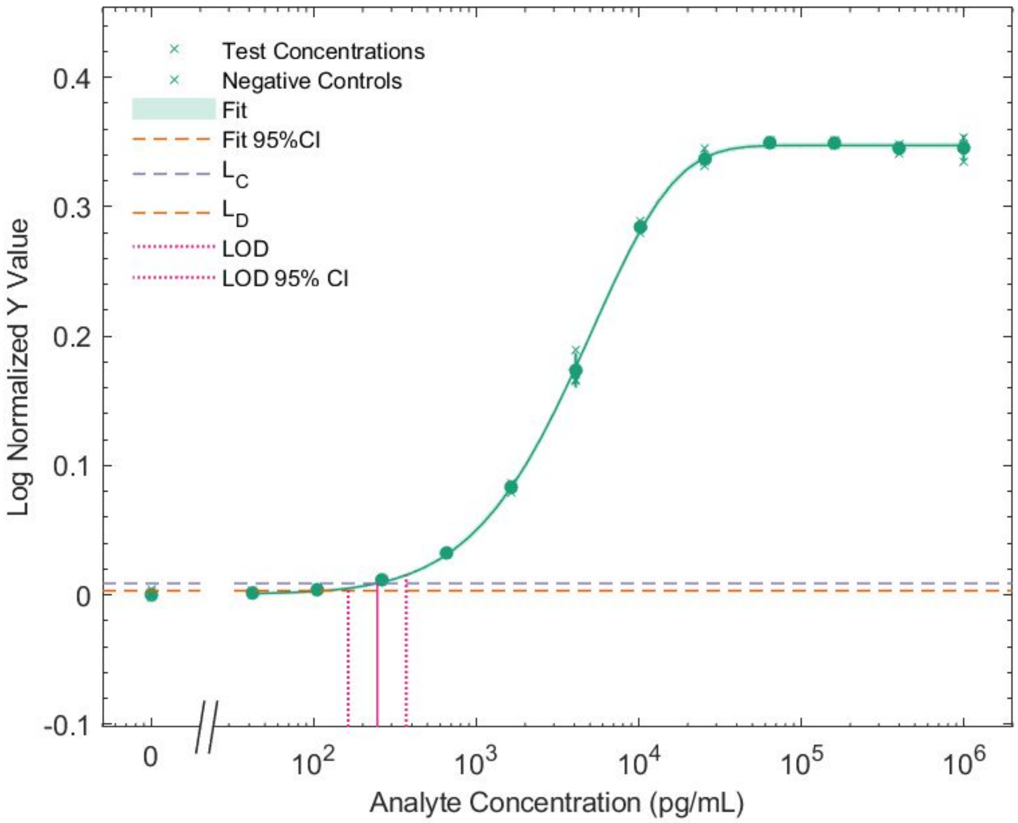

Figure S5: Capture B003, Detection 40588-MM124 sigmoidal regression curve, n = 3.

Table S11: Final Capture B003, Detection 40588-MM124 Detection Limit Fitting outputs.

Capture B003, Detection 40588-MM124 Detection Limit Fitting Outputs (5 parameter model)

Parameter	Value
**LOD**	245.7268
**LOD lower**	162.6477
**LOD upper**	370.7265
**Confidence level negatives**	0.05
**Confidence level positives**	0.05
**Confidence level variances**	0.05
**L_D**	0.0031461
**L_C**	0.0088383
**Units**	pg/mL

Capture B003, Detection 40588-MM124 Pair Checkerboard ELISA

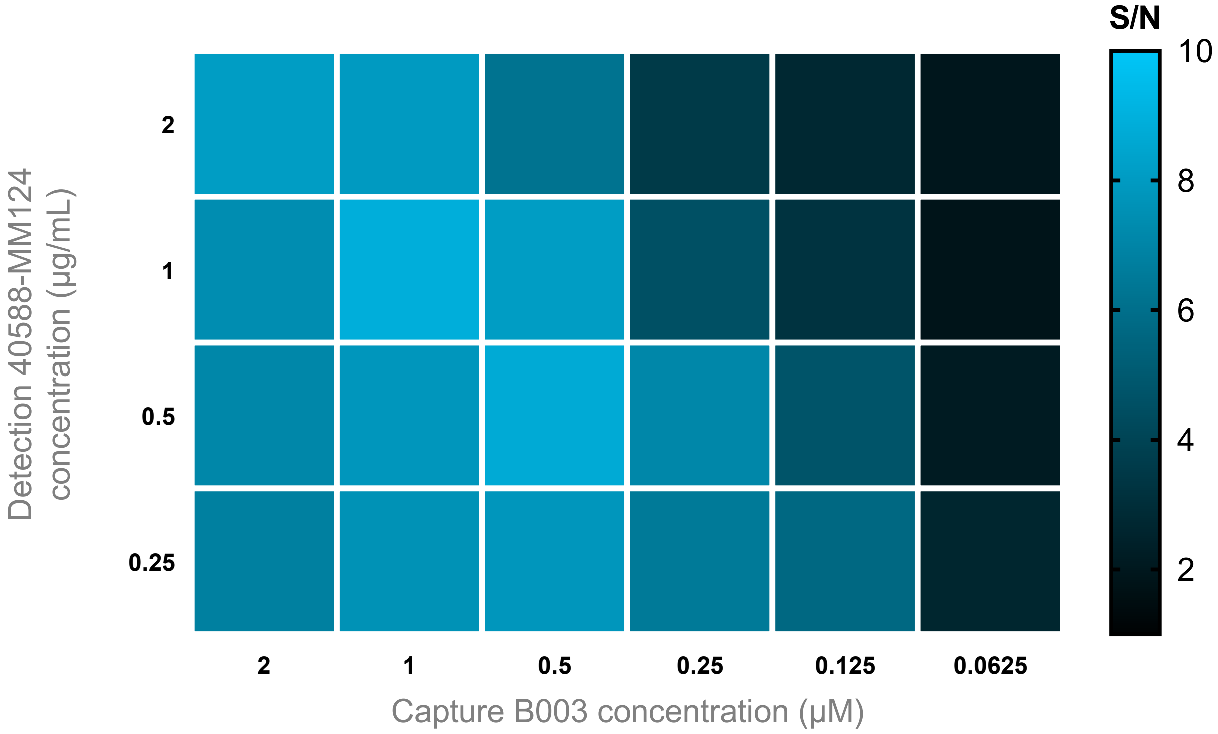

Figure S6: Capture B003, Detection 40588-MM124 Pair Checkerboard ELISA showing working capture & detection concentration performance.

AuNP seed characterisation

DLS

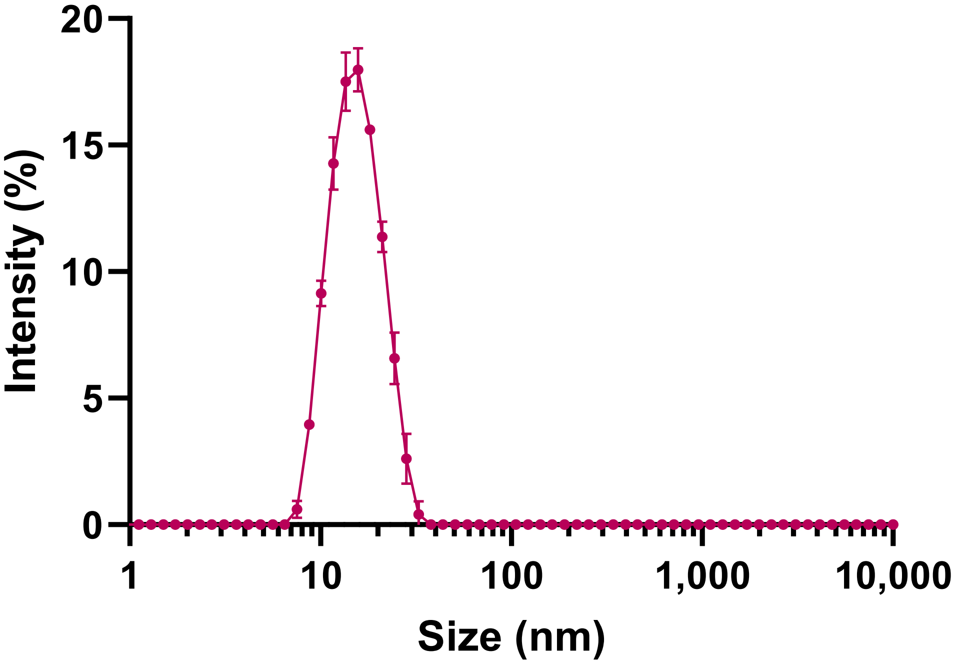

Figure S7: DLS showing intensity distribution of AuNP seed size for PtNC synthesis.

TEM

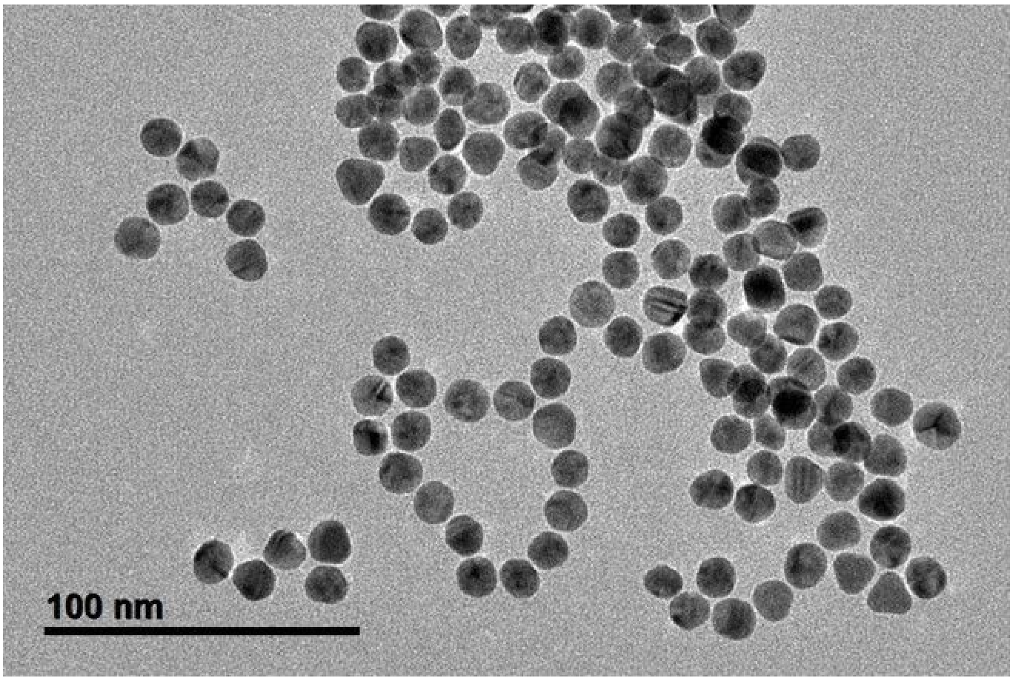

Figure S8: AuNP seed TEM.

PtNC Characterisation

DLS

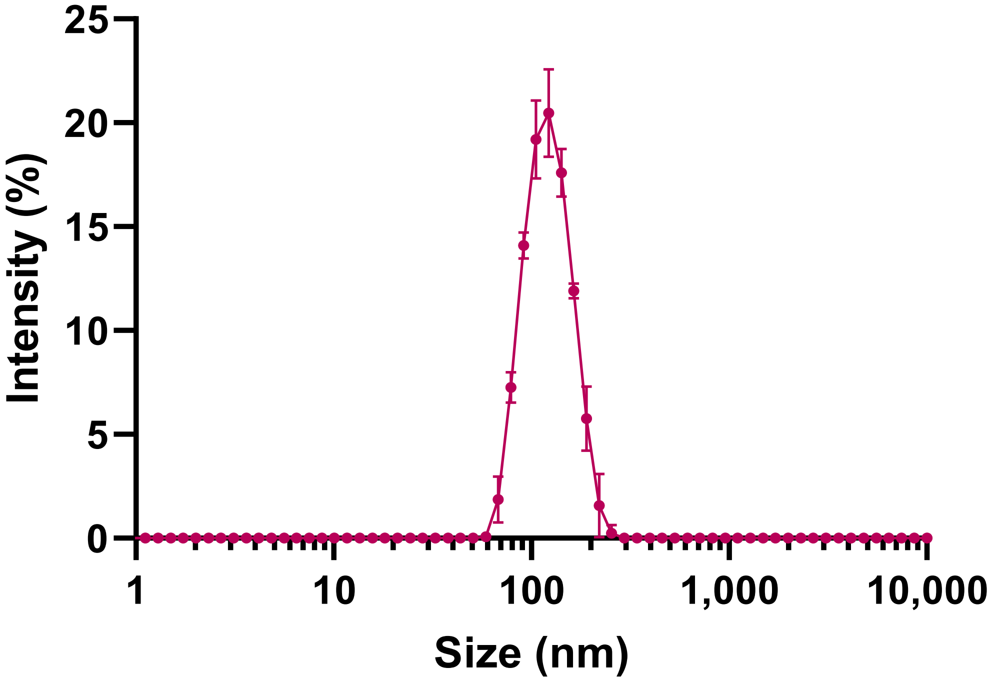

Figure S9: DLS showing intensity distribution of PtNC size.

TEM

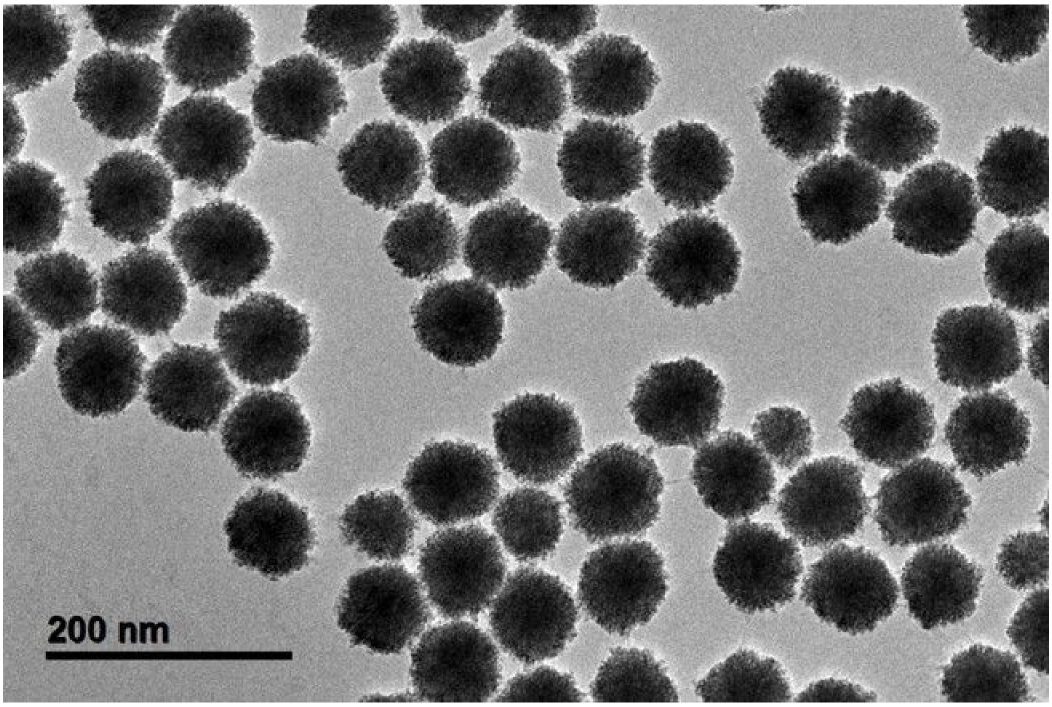

Figure S10: PtNC TEM.

PtNC Conjugation Characterisation

DLS

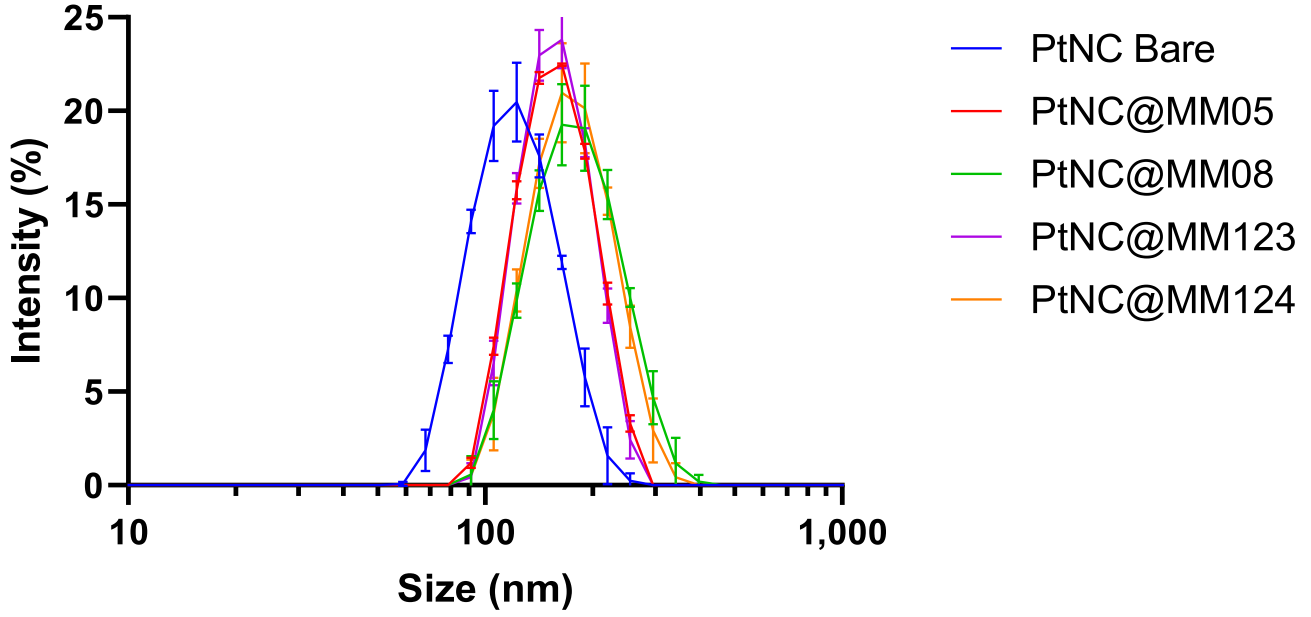

Figure S11: DLS showing intensity-based distribution of PtNC@Ab conjugates.

Table S12: Tabulated DLS & Zeta potential parameters of PtNC & PtNC@Ab conjugates, n = 3.

Sample Name	Z-Average (d.nm)	PdI	Intensity Mean (d.nm)	Number Mean (d.nm)	Volume Mean (d.nm)	Zeta Potential (mV)
PtNC Bare	116.7	0.057	124.7	96.02	125.5	-30.6
PtNC@40143-MM05	153.3	0.025	160.8	146.4	174.2	-32.1
PtNC@40143-MM08	171.7	0.073	185.2	164.8	209.1	-32.6
PtNC@40588-MM123	153.8	0.022	160.6	148.6	173	-32.1
PtNC@40588-MM124	168.9	0.044	179.4	162.3	198.8	-30.8

NLISA Heatmap SD

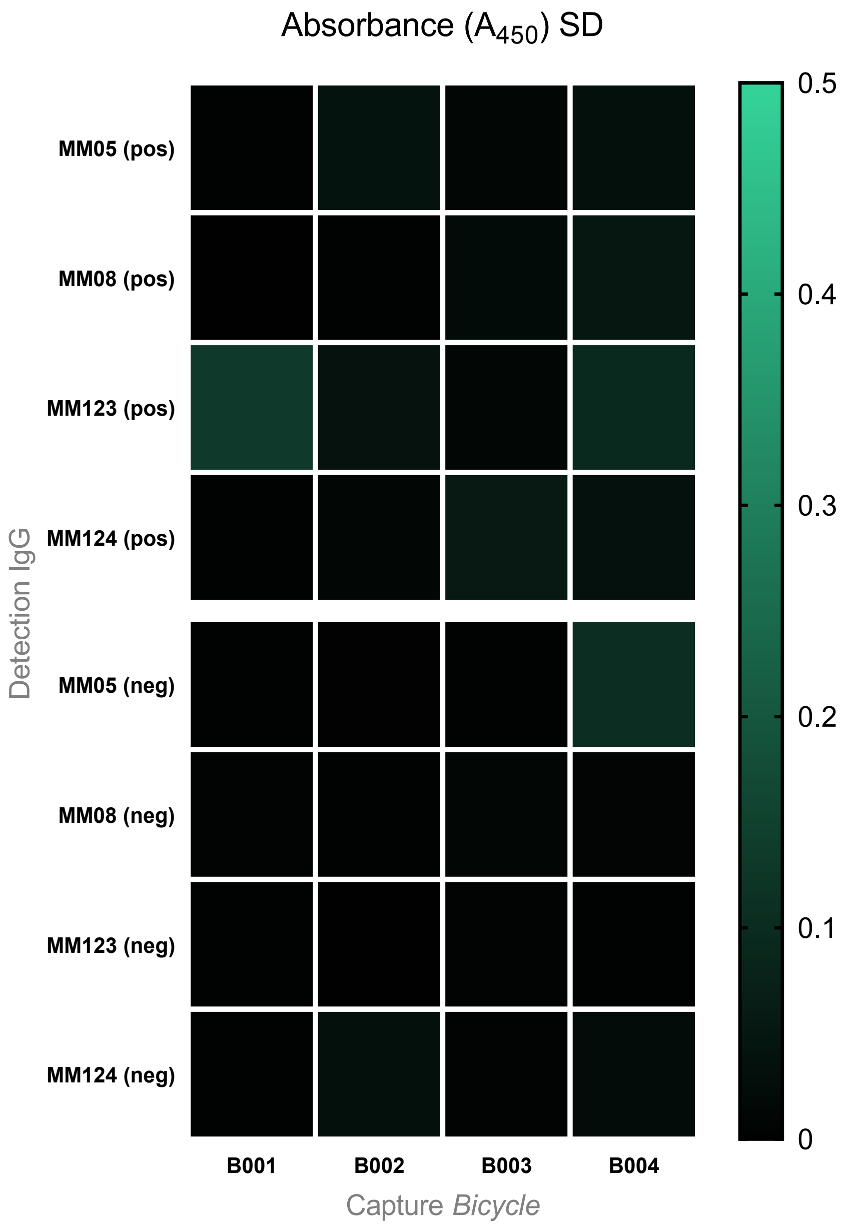

Figure S12: NLISA biorecognition pair standard deviation heatmap, n = 3.

NLISA Heatmap CV

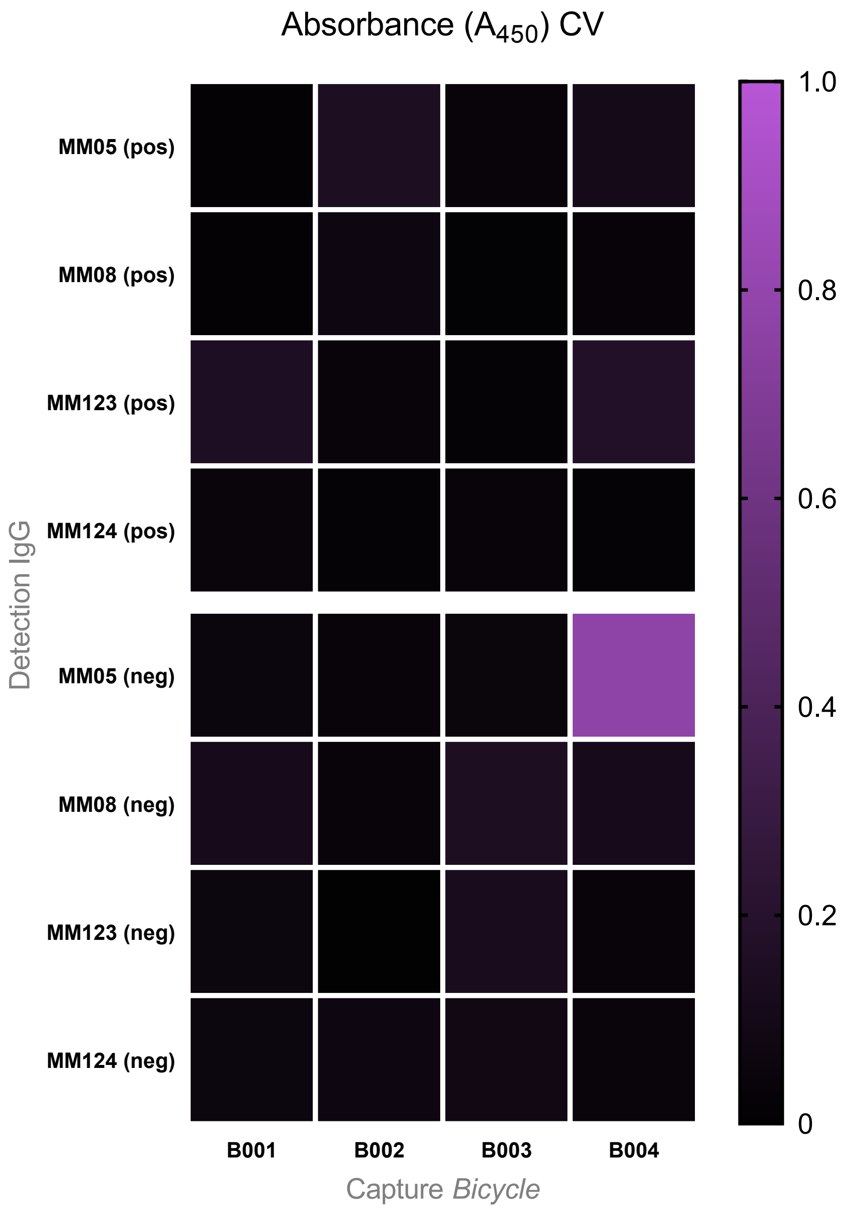

Figure S13: NLISA biorecognition pair standard deviation coefficient of variation, n = 3.

NLISA Pair Tabulated Parameters

Table S13: Capture B003, Detection 40143-MM08 Detection Limit Fitting Outputs.

Capture B003, Detection 40143-MM08 Detection Limit Fitting Outputs (5 parameter model)

Parameter	Value
**LOD**	109.8007
**LOD lower**	66.6362
**LOD upper**	180.111
**Confidence level negatives**	0.05
**Confidence level positives**	0.05
**Confidence level variances**	0.05
L_D	0.0019169
L_C	0.01251
**Units**	pg/mL

Table S14: Capture B003, Detection 40143-MM08 sigmoidal regression curve parameters. The data have been extracted from the four-parameter model used to fit the standard curve.

Capture B003, Detection 40143-MM08 Tabulated Parameters (4 parameter model)

Best-fit values	
**Bottom**	0.07535
**Hillslope**	1.437
**Top**	1.738
**EC50**	1.158
**R squared**	0.9974

Table S15: Capture B004, Detection 40143-MM08 Detection Limit Fitting Outputs.

Capture B004, Detection 40143-MM08 Detection Limit Fitting Outputs (5 parameter model)

Parameter	Value
**LOD**	94.1557
**LOD lower**	59.212
**LOD upper**	149.0476
**Confidence level negatives**	0.05
**Confidence level positives**	0.05
**Confidence level variances**	0.05
L_D	0.0045427
L_C	0.0091546
**Units**	pg/mL

Table S16: Capture B004, Detection 40143-MM08 sigmoidal regression curve parameters. The data have been extracted from the four-parameter model used to fit the standard curve.

Capture B004, Detection 40143-MM08 Tabulated Parameters (4 parameter model)

Best-fit values	
**Bottom**	0.08272
**Hillslope**	1.559
**Top**	1.691
**EC50**	0.9603
**R squared**	0.9972

Table S17: Capture B003, Detection 40588-MM124 Detection Limit Fitting Outputs.

Capture B003, Detection 40588-MM124 Detection Limit Fitting Outputs (5 parameter model)

Parameter	Value
**LOD**	119.9824
**LOD lower**	41.7443
**LOD upper**	338.1518
**Confidence level negatives**	0.05
**Confidence level positives**	0.05
**Confidence level variances**	0.05
L_D	0.0021966
L_C	0.0089341
**Units**	pg/mL

Table S18: Capture B003, Detection 40588-MM124 sigmoidal regression curve parameters. The data have been extracted from the four-parameter model used to fit the standard curve.

Capture B003, Detection 40588-MM124 Tabulated Parameters (4 parameter model)

Best-fit values	
**Bottom**	0.08188
**Hillslope**	1.336
**Top**	1.546
**EC50**	1.944
**R squared**	0.9892

NLISA Best Pairs ANOVA Test

Table S19: NLISA Best biorecognition pair ANOVA test (Tukey-Kramer post-hoc test)

Comparison 1	Comparison 2	Lower CI of log[LOD (pg/mL)] difference	CI of log[LOD (pg/mL)] difference	Upper CI of log[LOD (pg/mL)] difference	P-value
B003, 40143-MM08	B003, 40588-MM124	-5.71E-01	-3.79E-02	4.95E-01	9.84E-01
B003, 40143-MM08	B004, 40143-MM08	-4.67E-01	6.55E-02	5.98E-01	9.53E-01
B003, 40588-MM124	B004, 40143-MM08	-4.30E-01	1.03E-01	6.36E-01	8.87E-01

Capture B004, Detection 40143-MM08 Pair Calibration Curve

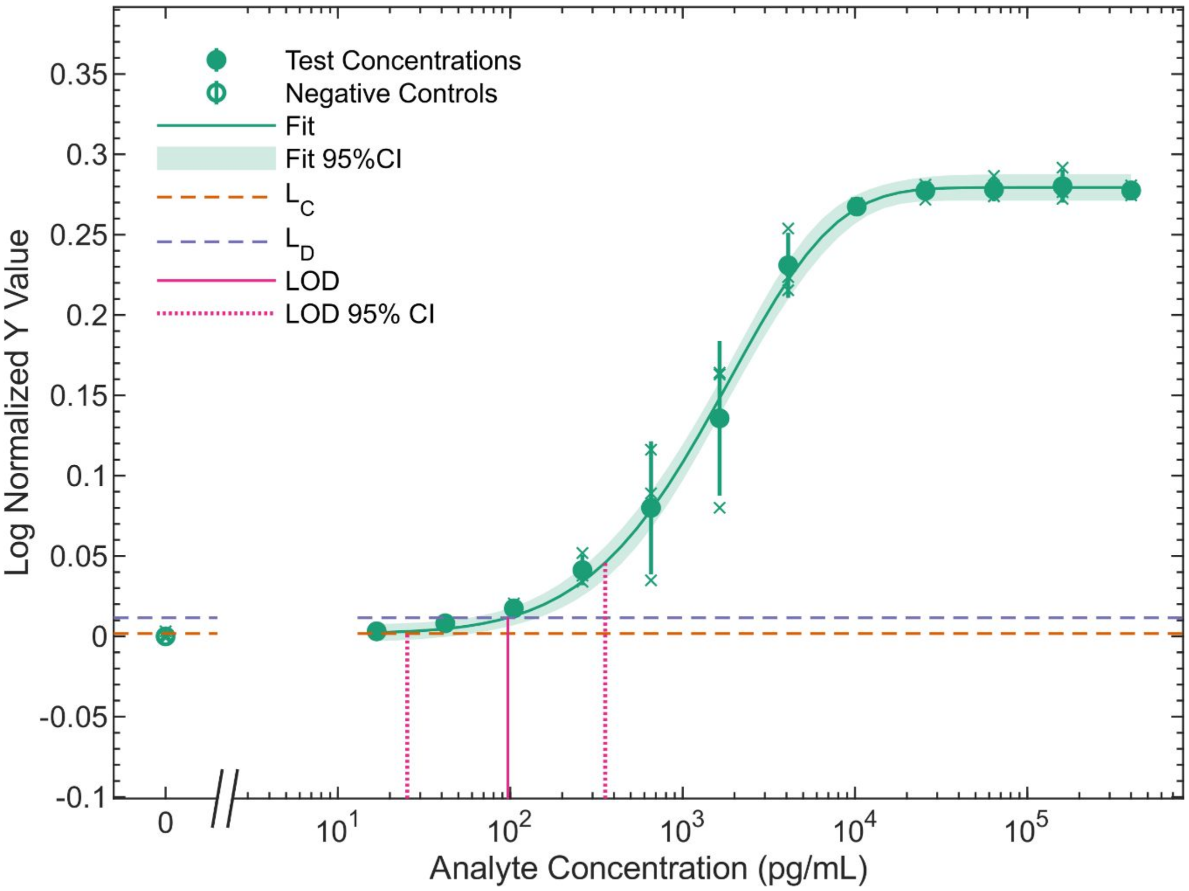

Figure S14: Capture B004, Detection 40143-MM08 sigmoidal regression curve, n = 3.

Table S20: Final Capture B004, Detection 40143-MM08 Detection Limit Fitting Outputs.

Parameter	Value
**LOD**	96.7139
**LOD lower**	25.2595
**LOD upper**	355.4701
**Confidence level negatives**	0.05
**Confidence level positives**	0.05
**Confidence level variances**	0.05
**L_D**	0.0017733
**L_C**	0.011581
**Units**	pg/mL

Capture B004, Detection 40143-MM08 Pair Checkerboard NLISA

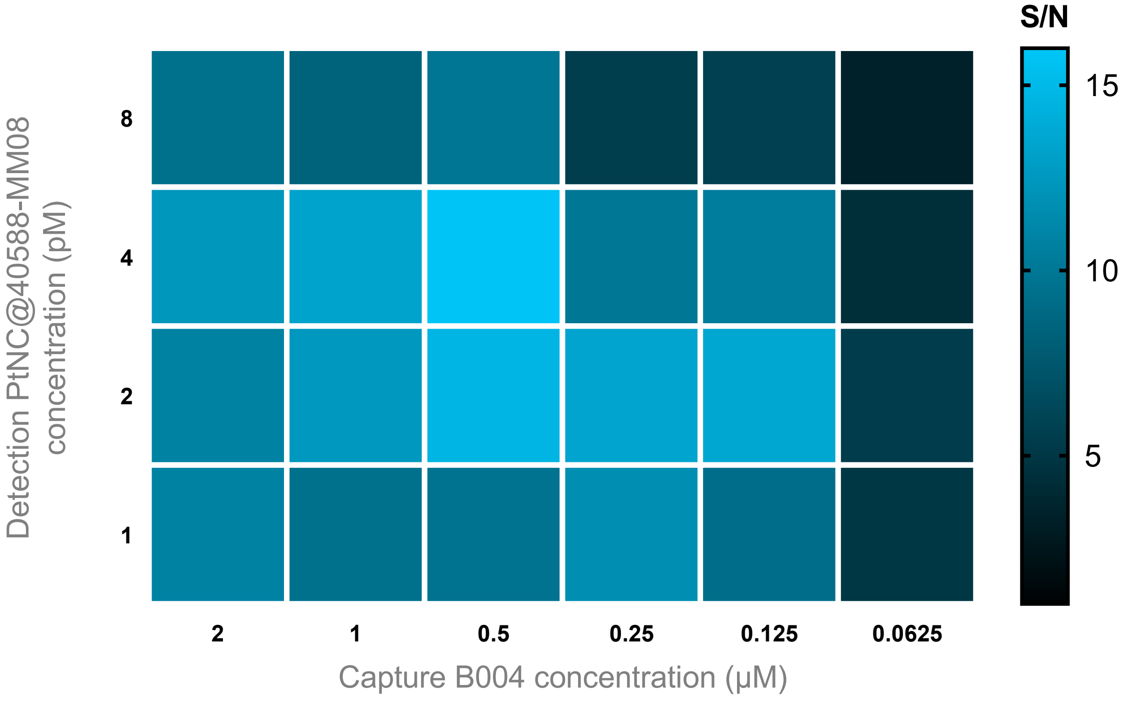

Figure S15: Capture B004, Detection 40143-MM08 Pair Checkerboard NLISA showing working capture & detection concentration performance.

LFIA Capture B001, Detection 40143-MM05 PtNC Calibration Curve Images

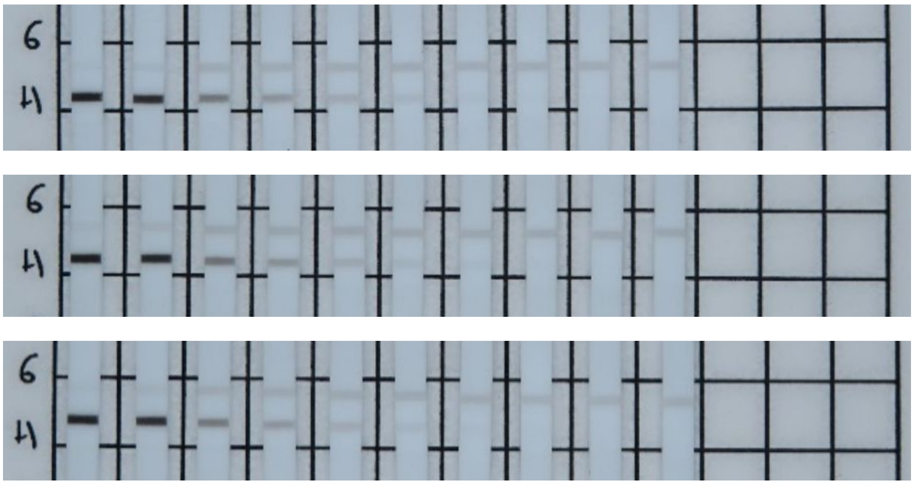

Figure S16: Pre-amplified calibration curve strips (100, 50, 10, 5, 2.5, 1.25, 0.2, 0.31, 0.16 & 0.08 ng/mL respectively shown).

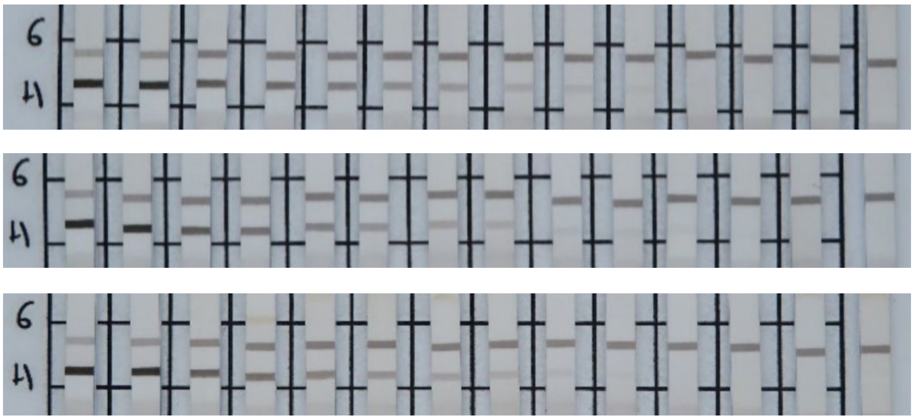

Figure S17: Amplified calibration curve strips (100, 50, 10, 5, 2.5, 1.25, 0.2, 0.31, 0.16, 0.08, 0.04, 0.02, 0.01 & 0 ng/mL respectively shown) after 5 min.

LFIA Capture B001, Detection 40143-MM05 AuNP Spiked FBST Calibration Curve Images

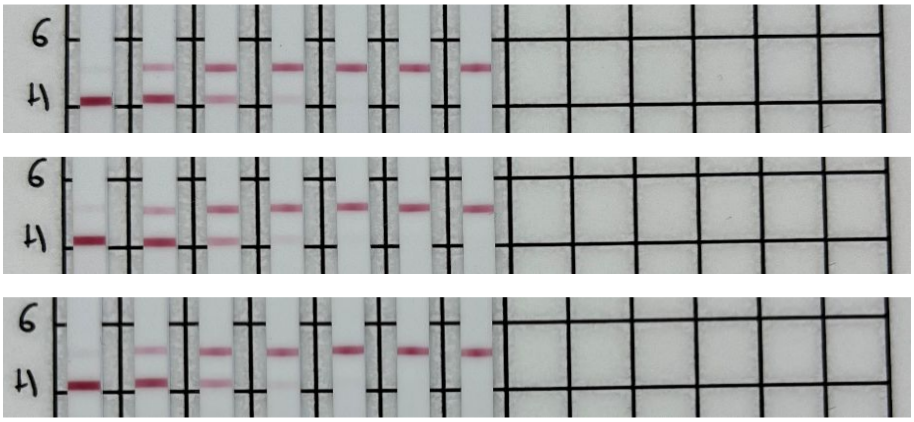

Figure S18: Calibration curve strips (500, 100, 20, 4, 0.8, 0.16 & 0 ng/mL respectively shown).

LFIA Capture B001, Detection 40143-MM05 AuNP Saliva Matrix Calibration Curve Images

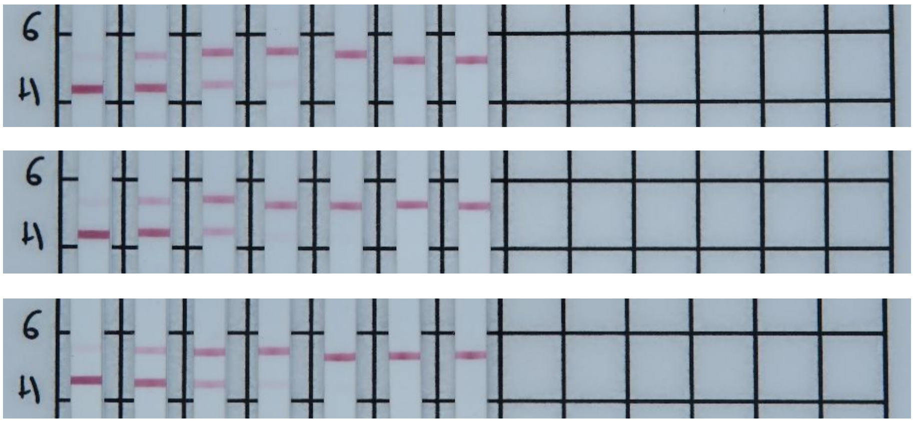

Figure S19: Calibration curve strips (500, 100, 20, 4, 0.8, 0.16 & 0 ng/mL respectively shown).
